# Regulation of Complement and Contact System Activation via C1 Inhibitor Potentiation and Factor XIIa Activity Modulation by Sulfated Glycans – Structure-Activity Relationships

**DOI:** 10.1371/journal.pone.0165493

**Published:** 2016-10-26

**Authors:** Ann-Kathrin Schoenfeld, Eric Lahrsen, Susanne Alban

**Affiliations:** Department of Pharmaceutical Biology, Pharmaceutical Institute, Christian-Albrechts-University of Kiel, Kiel, Schleswig-Holstein, Germany; University of Manchester, UNITED KINGDOM

## Abstract

The serpin C1 inhibitor (C1-INH) is the only regulator of classical complement activation as well as the major regulator of the contact system. Its importance is demonstrated by hereditary angioedema (HAE), a severe disease with potentially life-threatening attacks due to deficiency or dysfunction of C1-INH. C1-INH replacement is the therapy of choice in HAE. In addition, C1-INH showed to have beneficial effects in other diseases characterized by inappropriate complement and contact system activation. Due to some limitations of its clinical application, there is a need for improving the efficacy of therapeutically applied C1-INH or to enhance the activity of endogenous C1-INH. Given the known potentiating effect of heparin on C1-INH, sulfated glycans (SG) may be such candidates. The aim of this study was to characterize suitable SG by evaluating structure-activity relationships. For this, more than 40 structurally distinct SG were examined for their effects on C1-INH, C1s and FXIIa. The SG turned out to potentiate the C1s inhibition by C1-INH without any direct influence on C1s. Their potentiating activity proved to depend on their degree of sulfation, molecular mass as well as glycan structure. In contrast, the SG had no effect on the FXIIa inhibition by C1-INH, but structure-dependently modulated the activity of FXIIa. Among the tested SG, β-1,3-glucan sulfates with a M_r_ ≤ 10 000 were identified as most promising lead candidates for the development of a glycan-based C1-INH amplifier. In conclusion, the obtained information on structural characteristics of SG favoring C1-INH potentiation represent an useful elementary basis for the development of compounds improving the potency of C1-INH in diseases and clinical situations characterized by inappropriate activation of complement and contact system.

## Introduction

C1 inhibitor (C1-INH) is a member of the serpin family of protease inhibitors and the major regulator of several serine proteases of the human complement, contact and coagulation system [1,2]. These plasmatic cascade systems are closely linked to each other and as part of the innate immunity indispensable for an adequate immune response [2]. Incorrect regulation, however, causes inflammation and targeting of self-tissue and is involved in numerous diseases, including autoimmune diseases (e.g. systemic lupus erythematosus), ischemia/reperfusion syndrome, sepsis, age-related degenerative diseases, transplant rejection, atherosclerosis, diabetes, thrombotic microangiopathies, thromboembolic diseases, inflammatory diseases (e.g. rheumatoid arthritis), and cancer [2–4].

Within the complement system, C1-INH is one of several soluble and membrane-bound regulatory proteins, however, the only one inhibiting the initial activation of the complement system that can occur via three different pathways, i.e. (1) the classical pathway (CP) mainly initiated by antibody complexes, (2) the lectin pathway (LP), that is activated by carbohydrates or *N*-acetylated groups on microbial surfaces, and (3) the alternative pathway (AP) which is triggered by foreign surfaces and functions as so-called amplification loop [5].

C1-INH inactivates the initiating enzymes C1r and C1s of the CP [6] and the mannan-binding lectin associated serine proteases MASP-1 and MASP-2 of the LP by covalent bond formation [7,8]. Additionally, C1-INH is suggested to inhibit the AP by reversible binding of C3b and thus by a non-serpin mechanism [9]. C1-INH, therefore, diminishes the consequences of complement activation, including the generation of pro-inflammatory anaphylatoxins, especially C5a, and finally the formation of a membrane attack complex (MAC) that leads to lysis of foreign cells.

Besides its role in the complement system, C1-INH is also the primary regulator of the contact system by inactivating the contact system / intrinsic coagulation proteases plasma kallikrein, factor XIIa (FXIIa), and factor XIa (FXIa) [10]. In this way it regulates the generation of bradykinin as well as the intrinsic coagulation pathway. Moreover, C1-INH was found to exhibit inhibitory effects also on other proteases including thrombin, plasmin, and tissue plasminogen activator [1].

Altogether, C1-INH contributes to the cross-linking between complement system, contact system, coagulation, and fibrinolysis [10].

The physiological importance of C1-INH is reflected in its association with various diseases including hereditary and acquired angioedema (HAE, AAE) with potentially life-threatening attacks of subcutaneous and submucosal edemas [11], and age-related macular degeneration (AMD) being the leading cause of blindness in the elderly in developed countries [12]. In HAE, mutations in the SERPING1 gene encoding C1-INH (HAE type I or II) [13] and in the FXII gene (HAE type III) [14,15] lead to insufficient regulation of the kallikrein/kinin system and thus to elevated bradykinin levels causing the typical symptoms like swelling of soft tissues due to its function as potent vasodilator [16,17].

C1-INH replacement with plasma-derived C1-INH concentrates (Berinert^®^P, Cinryze^®^) is still the therapy of choice in both acute management and prophylactic treatment of HAE [18]. Only for treatment of acute attacks, three further drugs have been approved in recent years: conestat alfa (Ruconest^®^), a recombinant human C1-INH obtained from milk of transgenic rabbits, icatibant (Firazyr^®^), a decapeptide blocking the bradykinin B2 receptor and the recombinant kallikrein inhibitor ecallantide (Kalbitor^®^), a recombinant protein selectively inhibiting kallikrein (for safety reasons not available in EU) [18–20]. However, the use of both C1-INH and the other drugs has limitations [21–23]: high costs of therapy, limited resources of human plasma in the case of plasma-derived C1-INH, risk of immunological side effects, and finally only partly allowed, challenging self-administration.

These may also be obstacles to the application of C1-INH in further indications, although clinical studies showed beneficial effects of C1-INH also in other disease states like sepsis, gram-negative endotoxic shock, vascular leak syndrome, transplant rejection, ischemia-reperfusion injury, myocardial infarction, and emergency coronary artery bypass surgery [24,25].

Consequently, there is still a need for improving the C1-INH replacement therapy and for alternatives to C1-INH. At this point, the potentiating effect of heparins and some other sulfated compounds on C1-INH, which is known for a long time [26], leads to the idea that sulfated glycans (SG) may be candidates to enhance the efficacy of therapeutically applied C1-INH or to increase the activity of endogenous C1-INH.

The C1-INH potentiating effect of unfractionated heparin (UFH) [26] was confirmed *in vivo* by increased C1rs-C1-INH complexes in the blood of patients undergoing coronary artery bypass grafting using heparin-coated surfaces [27]. Furthermore, a limited number of other SG was analyzed *in vitro*, yet, with varying results. In one study dextran sulfates (DexS) with different molecular masses potentiated the C1s inhibition by C1-INH even stronger than UFH [28], whereas others found a C1-INH potentiating effect smaller than that of UFH [26]. The glycosaminoglycans (GAGs) heparan sulfate (HS) and chondroitin sulfate (CS) were consistently less active than UFH or even inactive [28–30], whereas oversulfated chondroitin sulfate (OSCS) was shown to increase the binding of C1-INH to C1s stronger than UFH [30]. Furthermore, a fucoidan extracted from the brown alga *Ascophyllum nodosum* turned out to influence the C1s inhibition by C1-INH only to a small extent [31]. Rossi et al. demonstrated that the C1-INH potentiation by heparin oligomers is due to binding to both C1-INH and C1s [32]. Just recently, the effects of UFH and low-molecular weight heparins (LMWHs) on CP, LP and AP activation in absence and presence of C1-INH were compared [33].

Investigations on the contact system proteases targeted by C1-INH revealed that UFH, HS, DexS, dermatan sulfate and LMWHs enhanced the inhibition of FXIa by C1-INH, but had no effect on kallikrein and even protected FXIIa from inhibition by C1-INH [34–36]. In contrast, Gozzo et al. found a slightly increased kallikrein inhibition by C1-INH in presence of UFH, HS and CS [37].

In view of these limited data, we examined more than 40 SG for their potentiating effect on the C1s inhibition by C1-INH. By evaluating structure-activity relationships, we aimed to characterize the structural features of SG principally suitable for use as C1-INH amplifier. Moreover, the structure-dependent effects of SG on FXIIa and its inhibition by C1-INH were investigated, since this key serine protease of the contact system additionally triggers the CP complement activation and is involved in the pathophysiology of HAE [38,11].

Besides various heparins and further glycosaminoglycans, native and partially depolymerized SG isolated from four different algae were included in the study. Two series of semi-synthetic, structurally defined β-1,3-glucan sulfates enabled us to examine the impact of molecular mass (M_r_) and degree of sulfation (DS) in more detail independently of the basic glycan structure.

The study revealed that the potentiating effect of SG on the C1s inhibition by C1-INH depends on their degree of sulfation (DS), their molecular mass (M_r_) as well as their glycan structure, whereby the impact of the M_r_ is closely linked to the DS of the SG. In contrast, the SG had no effect on the inhibition of FXIIa by C1-INH, but structure-dependently either stimulated or inhibited the activity of FXIIa directly.

## Materials and Methods

### Materials

C1 inhibitor from human plasma (C1-INH, GenWay Biotech, San Diego, USA) was diluted with phosphate buffer (20 mmol/l KH_2_PO_4_, 250 mmol/l KCl, pH 7.0) to a 5 mg/ml stock solution, aliquoted and stored at -80°C. The Technochrom^®^ C1-INH test kit (Technoclone, Vienna, Austria) provided C1s esterase, a chromogenic substrate for C1s (C_2_H_5_CO-Lys(ϵ-Cbo)-Gly-Arg-pNA) and ready-to-use buffers (TRIS buffer A: 50 mmol/l TRIS, 257 mmol/l NaCl, pH 7.4; TRIS buffer B: 50 mmol/l TRIS, 257 mmol/l NaCl, pH 8.3). According to the product descriptions, both lyophilized enzyme and substrate were reconstituted with Ampuwa^®^.

Human α-Factor XIIa (FXIIa, Enzyme Research Laboratories, South Bend, USA) was diluted with sodium acetate buffer (4 mmol/l sodium acetate, 150 mmol/l NaCl, pH 5.3) to a 1 mg/ml stock solution, aliquoted and stored at -80°C.

The chromogenic substrate for FXIIa (S-2302^TM^, H-D-Pro-Phe-Arg-pNA • 2 HCl) was obtained from Chromogenix (Instrumentation Laboratory, Bedford, USA) and diluted with Ampuwa^®^ to a 4 mmol/l stock solution.

### Test compounds

Structural characteristics of all test compounds including their molecular mass (M_r_) and degree of sulfation (DS) are indicated in Table 1. All anionic glycans were used in form of the sodium salt, if not indicated otherwise.

**Table 1:** Structural characteristics of the tested compounds.

Test compound	Abbreviation	M_r_^1^	DS^2^
**Heparins and modified heparins**			
Unfractionated heparin	UFH	15 000 ^a^	1.00 ^e^
Medium-molecular weight heparin	MMWH	10 500 ^a^	1.00 ^e^
Tinzaparin	TINZA	6 500 ^b^	1.00 ^e^
Certoparin	CERTO	5 200 ^b^	1.00 ^e^
Enoxaparin	ENOXA	4 500 ^b^	1.00 ^b^
Nadroparin	NADRO	4 300 ^b^	1.00 ^b^
Very-low-molecular weight heparin	VLMWH	3 400 ^a^	1.00 ^e^
Pentasaccharide fragments	PENTA	1 500 ^a^	1.00 ^e^
Fondaparinux	FPX	1 728 ^a^	1.60 ^a^
De-*N*-sulfated UFH	de*N*sUFH	15 000 ^a^	0.50 ^a^
*N*-acetyl-de-*O*-sulfated UFH	de*O*sUFH	15 000 ^a^	<0.10 ^a^
Oversulfated heparin	OSHep	12 500 ^a^	1.75 ^a^
**Glycosaminoglycans**			
Chondroitin sulfate A	CS-A	18 600 ^c^	0.30 ^e^
Chondroitin sulfate B	CS-B	21 000 ^c^	0.38 ^e^
Chondroitin sulfate C	CS-C	71 800 ^c^	0.37 ^e^
Oversulfated chondroitin sulfate	OSCS	16 600 ^c^	2.00 ^e^
Heparan sulfate 1	HS33-04	33 400 ^c^	0.42 ^a^
Heparan sulfate 2	HS11-08	10 800 ^c^	0.80 ^a^
Danaparoid sodium	DANA	6 000 ^a^	0.50 ^e^
Hyaluronic acid	HA	915 000 ^c^	0.00 ^f^
Hyaluronic acid oligosaccharides	oligoHA	3 600 ^a^	0.00 ^f^
**Dextrans and dextran sulfates**			
Low-molecular mass dextran	Dex-L	32 000 ^a^	-
High-molecular mass dextran	Dex-H	5 000 000 ^a^	-
Low-molecular mass dextran sulfate	DexS-L	5 000 ^a^	2.02 ^e^
High-molecular mass dextran sulfate	DexS-H	500 000 ^a^	1.54 ^e^
**β-1,3-glucan sulfates**			
Curdlan sulfate 1	CurS1	210 000 ^c^	0.64 ^g^
Curdlan sulfate 2	CurS2	160 000 ^c^	1.34 ^g^
Curdlan sulfate 3	CurS3	160 000 ^c^	1.74 ^g^
Curdlan sulfate 3a	CurS3a	70 000 ^c^	1.78 ^g^
Phycarin sulfate 1	PhyS1	6 000 ^c^^,^ ^d^	0.75 ^g^
Phycarin sulfate 2	PhyS2	8 000 ^c^^,^ ^d^	1.48 ^g^
Phycarin sulfate 3	PhyS3	9 000 ^c^^,^ ^d^	1.80 ^g^
Phycarin sulfate 4	PhyS4 (PS3)	10 000 ^c^^,^ ^d^	2.21 ^g^
Phycarin sulfate 5	PhyS5	12 000 ^c^^,^ ^d^	3.00 ^g^
**Algae-derived sulfated polysaccharides**			
*Saccharina latissima*-SP batch 1	*S*.*l*.-SP#1	534 000 ^c^	0.58 ^e^
*Saccharina latissima*-SP batch 2	*S*.*l*.-SP#2	541 000 ^c^	0.35 ^e^
*Fucus vesiculosus*-SP batch a	*F*.*v*.-SP#a	27 000 ^c^	0.53 ^e^
*Fucus vesiculosus*-SP batch b	*F*.*v*.-SP#b	38 000 ^c^	0.63 ^e^
*Delesseria sanguinea*-SP	*D*.*s*.-SP	142 000 ^c^	0.71 ^e^
*Coccotylus truncatus-*SP	*C*.*t*.*-*SP	128 000 ^c^	0.47 ^e^
Sodium alginate	ALG	120 000 ^a^	0.00 ^f^

^1^ relative molecular mass

^2^ degree of sulfation (sulfate groups per monosaccharide in relation to the absolute glycan content)

^a^ according to the product specification / certificate of analysis

^b^ according to the European Pharmacopoeia (Ph. Eur.)

^c^ determined by size exclusion chromatography with a MALLS detector; the resulting molecular mass is the weight averaged M_r_ (M_w_)

^d^ degree of polymerization of about 25; different M_r_ result from different degrees of sulfation

^e^ calculated from sulfur content (elemental analysis)

^f^ negatively charged due to uronic acid content

^g^ determined by ion chromatography (HPLC)

#### Heparins

Several heparins and modified heparins were tested: Besides unfractionated heparin from porcine intestinal mucosa (UFH, 200 IU/mg, Novartis, Nürnberg, Germany), the low-molecular-weight heparins (LMWHs) (1) nadroparin (NADRO, GlaxoSmithKline, München, Germany), (2) enoxaparin (ENOXA, BfArM sample no. 11-07/08 05.06.2008), (3) certoparin (CERTO, Novartis, Nürnberg, Germany), and (4) tinzaparin (TINZA, EDQM, Strasbourg, France) were included, as well as *N*-acetyl-de-*O*-sulfated heparin (de*O*sUFH, Sigma-Aldrich, St. Louis, Missouri, USA) de-*N*-sulfated heparin (de*N*sUFH, Sigma-Aldrich, St. Louis, Missouri, USA) and oversulfated heparin (OSHep, Neoparin, Alameda, California, USA). Medium-molecular weight heparin (MMWH), very-low-molecular weight heparin (VLMWH), and oligosaccharides mainly consisting of pentasaccharide units (PENTA) were kind gifts from Novartis. Fondaparinux (FPX, Arixtra^®^) was a kind donation from Glaxo Smith Kline (Notre Dame de Bondville, France).

#### Other glycosaminoglycans

A series of both naturally occurring and chemically modified glycosaminoglycans (GAGs) was included in the study: Chondroitin sulfate A from bovine trachea (CS-A, Sigma-Aldrich, St. Louis, Missouri, USA), chondroitin sulfate B from porcine intestinal mucosa, synonym dermatan sulfate (CS-B, Sigma-Aldrich, St. Louis, Missouri, USA), and chondroitin sulfate C from shark cartilage (CS-C, Sigma-Aldrich, St. Louis, Missouri, USA) were included in the study.

Oversulfated chondroitin sulfate (OSCS) was synthesized by sulfation of chondroitin-4-sulfate (CS-A, as described above) according to Maruyama et al. [39].

Two different heparan sulfates were tested and compared with danaparoid: (1) heparan sulfate isolated from bovine kidney (HS33-04, Sigma Aldrich, St. Louis, Missouri, USA), (2) heparan sulfate isolated from porcine intestinal mucosa (HS11-08, Iduron, Cheshire, UK).

Danaparoid (DANA, EDQM, Strasbourg, France) is a complex mixture of sulfated GAGs prepared from porcine intestinal mucosa. Its major constituents are heparan sulfate, dermatan sulfate (8–16% (m/m)) and diverse types of chondroitin sulfates (up to 8.5% (m/m)) [40].

Hyaluronic acid (HA, potassium salt, Merck KGaA, Darmstadt, Germany) and hyaluronic acid fragments with a degree of polymerization of about 18 (oligoHA, Iduron, Chesire, UK) were chosen as non-sulfated GAGs.

#### Dextrans and dextran sulfates

We tested a low-molecular mass dextran (Dex-L, SERVA Electrophoresis, Heidelberg, Germany) and a high-molecular mass dextran (Dex-H, SERVA Electrophoresis, Heidelberg, Germany) as well as a low-molecular mass dextran sulfate (DexS-L, Sigma-Aldrich, St. Louis, Missouri, USA) and a high-molecular mass dextran sulfate (DexS-H, SERVA Electrophoresis, Heidelberg, Germany).

#### β-1,3-glucan sulfates

Two series of semisynthetic β-1,3-glucan sulfates differing in their M_r_ and DS were studied. Both series were produced by sulfation of natural linear β-1,3-glucans with SO_3_/pyridine in dimethylformamide: (1) curdlan sulfates (CurS) are derivatives of the high-molecular mass curdlan (Wako Pure Chemicals Industries, Osaka, Japan) [41], (2) phycarin sulfates (PhyS) were synthesized using the low-molecular mass Phycarin^®^ (Goëmar Laboratories, St. Malo, France) as starting material [42,43].

#### Algae-derived sulfated glycans and sodium alginate

The fucose-containing sulfated polysaccharides from *Saccharina latissima* (*S*.*l*.-SP) were extracted from dried material of the brown alga *Saccharina latissima* L. as previously described and identified as sulfated galactofucan with about 60% fucose and 15% galactose as main monosaccharides [44]. We examined two batches deriving from the North Atlantic Ocean harvested either in May (*S*.*l*.-SP#2) or in September (*S*.*l*.-SP#1). The two batches mainly differ in their DS, since both habitat and harvest time have an impact on the sulfate content of the *S*.*l*.-SP [44].

Further, two batches of the commercially available fucose-containing sulfated polysaccharides from *Fucus vesiculosus* L., named fucoidan and containing about 82% fucose, (*F*.*v*.-SP#a/b, Sigma-Aldrich, St. Louis, Missouri, USA) were included in the study.

Sulfated polysaccharides from the red algae *Delesseria sanguinea* (Hudson, Lamouroux) (*D*.*s*.-SP) and *Coccotylus truncatus* (*C*.*t*.-SP) were isolated using a standardized procedure and characterized as homogeneous fractions of branched sulfated xylogalactans, with about 76% galactose and 13% xylose in case of *D*.*s*.-SP and about 87% galactose and 4% xylose in case of *C*.*t*.-SP [45].

Additionally, we included series of *S*.*l*.-SP, *F*.*v*.-SP and *C*.*t*.*-*SP samples, which had been subjected to partial hydrothermal depolymerization (Table 2).

**Table 2:** C1 inhibitor potentiation of partially depolymerized algae-derived sulfated glycans.

Depolymerization time at 120°C	M_r_^1^	DS^2^	C1 inhibitor potentiation (%)^3^
***Saccharina latissima* sulfated glycans *S*.*l*.-SP#2**			
0 min	541 000	0.35	53.1 ± 3.8
10 min	325 000	0.35	53.2 ± 3.1
30 min	218 000	0.35	52.3 ± 4.3
60 min	151 000	0.35	48.4 ± 3.3
90 min	129 000	0.35	45.7 ± 2.1
160 min	77 000	0.35	41.4 ± 1.4
***Fucus vesiculosus* sulfated glycans *F*.*v*.-SP#b**			
0 min	38 000	0.63	24.8 ± 2.1
10 min	34 400	0.63	25.8 ± 2.1
20 min	29 600	0.63	26.7 ± 1.9
30 min	24 000	0.63	25.7 ± 2.3
40 min	20 600	0.63	26.3 ± 1.7
60 min	15 900	0.63	20.8 ± 1.4
90 min	10 300	0.63	9.9 ± 1.7
***Coccotylus truncatus* sulfated glycans *C*.*t*.-SP**			
0 min	128 000	0.47	14.8 ± 0.7
90 min	73 000	0.47	13.7 ± 2.0

^1^ relative molecular mass; determined by size exclusion chromatography with a MALLS detector; the indicated molecular mass is the weight averaged M_r_ (M_w_)

^2^ degree of sulfation (sulfate groups per monosaccharide in relation to the absolute glycan content) calculated from sulfur content (elemental analysis)

^3^ test compounds in a final concentration of 6.25 μg/ml, mean ± SD, n = 4 test runs on different days

Sodium alginate was used as representative of non-sulfated algae-derived glycans (ALG, Sigma-Aldrich, St. Louis, Missouri, USA).

### Methods

#### Testing of the C1s inhibition by C1 inhibitor and the influence of sulfated glycans

The effect of C1-INH on the activity of C1s esterase in the presence and absence of test compounds was measured by a chromogenic substrate assay performed in microplates (nunc™ 269620, Thermo Fisher Scientific, Langenselbold, Germany).

The C1-INH stock solution was diluted with TRIS buffer A (50 mmol/l TRIS, 257 mmol/l NaCl, pH 7.4) to a working concentration of 15 μg/ml (143 nmol/l). C1s esterase was diluted 1:4 with TRIS buffer A and stored on ice until use. The chromogenic substrate was diluted 1:6 with TRIS buffer B (50 mmol/l TRIS, 257 mmol/l NaCl, pH 8.3). The test compounds were diluted to the working concentration of 50 μg/ml with veronal buffer saline (VBS, 4.94 mmol/l 5,5-diethylbarbituric acid, 145 mmol/l NaCl, 0.83 mmol/l magnesium chloride, 0.25 mmol/l calcium chloride dihydrate, pH 7.3). Moreover, dilution series of the following compounds were prepared in VBS: UFH, PS3, OSHep: 0.1, 1.0, 10.0, 50.0, 100.0 μg/ml; CurS and PhyS series: 2.5, 5.0, 10.0, 50.0 μg/ml.

Initially, the microplate was loaded with 20 μl sample solution per well and incubated at 37°C for 10 min. Then, 20 μl of each, C1s and C1-INH were added. After incubation at 37°C under shaking for 5 min, 100 μl of prewarmed chromogenic substrate (final concentration: 0.625 mmol/l) were pipetted into each well. The plate was further incubated at 37°C under shaking for 15 min. The reaction was stopped by addition of 40 μl acetic acid 50% per well. The absorbance was measured at 405 nm against blank. Control values were (1) VBS instead of test compound and TRIS buffer A instead of C1-INH (100% C1s activity), (2) VBS instead of test compound (100% C1-INH potency) and (3) VBS instead of test compound, TRIS buffer A instead of C1-INH and C1s (blank). All samples and controls of a test run were tested in duplicate on each microplate and each test run was repeated at least three times on different days.

#### Testing of direct influences of sulfated glycans on the C1s activity

According to the procedure described above, the direct influence of all test compounds on the C1s activity was checked. For this, C1-INH was replaced by 20 μl TRIS buffer A. All samples were tested in duplicate per microplate on at least three different days.

#### Testing of the FXIIa inhibition by C1 inhibitor and the influence of sulfated glycans

The effect of C1-INH on the activity of FXIIa in the presence and absence of UFH, PS3, CurS3 and *S*.*l*.-SP#1 was measured by a chromogenic substrate assay performed in microplates (nunc™ 269620, Thermo Fisher Scientific, Langenselbold, Germany).

The C1-INH stock solution was diluted with TRIS buffer (50 mmol/l TRIS, 113 mmol/l NaCl, pH 7.8) to a working concentration of 20 μg/ml (191 nmol/l). FXIIa was diluted with TRIS buffer to a working concentration of 30 μg/ml (375 nmol/l) and stored on ice until use. The test compounds were diluted to the working concentration with veronal buffer saline (VBS, 4.94 mmol/l 5,5-diethylbarbituric acid, 145 mmol/l NaCl, 0.83 mmol/l magnesium chloride, 0.25 mmol/l calcium chloride dihydrate, pH 7.3).

Initially, the microplate was loaded with 20 μl sample solution per well and incubated at 37°C for 10 min. Then, 20 μl of each, FXIIa and C1-INH were added. After incubation at 37°C under shaking for 10 min, 100 μl of S-2302 1 mmol/l in TRIS buffer (50 mmol/l TRIS, pH 7.8) were pipetted into each well. The kinetics of the absorbance at 405 nm was measured at 37°C for 30 min, whereby the data points after 10 min served as values to compare the test compounds. Control values were (1) VBS instead of test compound and TRIS buffer instead of C1-INH (100% FXIIa activity), (2) VBS instead of test compound (100% C1-INH potency) and (3) VBS instead of test compound, TRIS buffer instead of C1-INH and FXIIa (blank). All samples were tested in duplicate per microplate.

#### Testing of direct influences of sulfated glycans on the FXIIa activity

According to the procedure described above, the direct influence of the test compounds on the FXIIa activity was examined. For this, the C1-INH was replaced by 20 μl TRIS buffer. All samples were tested in duplicate per microplate on at least two different days.

#### Data analysis

Linear and non-linear regressions and statistics were calculated using SigmaPlot^TM^ 11.0 (Systat Software Inc.). P-values of ≤ 0.05 were considered significant and were calculated with a one-way analysis of variance followed by multiple comparisons versus control (Holm-Sidak method) unless stated otherwise.

## Results

### Evaluation of incubation times in the C1-INH/C1s assay

In the course of establishing the C1-INH/C1s assay, the effect of various incubation procedures was examined, whereby PS3 was used as exemplary C1-INH amplifier. The aims were (1) to determine the extent and velocity of C1s inhibition by C1-INH, (2) to estimate the extent of C1-INH potentiation by PS3, and (3) to evaluate the influence of preincubation of PS3 with C1-INH alone as well as of incubation with both C1-INH and C1s on its C1-INH potentiating effect.

#### Influence of preincubation of test compound and C1 inhibitor on C1s inhibition

After a preincubation of C1-INH in absence and presence of PS3 (final concentration: 0.625 μg/ml) for 0, 2, 5, 10 or 15 min at 37°C the residual C1s activity was determined as described in materials and methods. The C1s inhibition was calculated in relation to 100% C1s activity without C1-INH and PS3.

C1-INH inhibited the amidolytic activity of C1s by 31 ± 1%. PS3 (final concentration: 0.625 μg/ml) increased this C1s inhibition by about 90 ± 10%. Preincubation of PS3 and C1-INH did not result in increased C1s inhibition. Therefore, the assay was subsequently performed without preincubation of C1-INH with test compound.

#### Influence of the incubation time of test compound with C1 inhibitor and C1s on C1s inhibition

**Fig 1:**
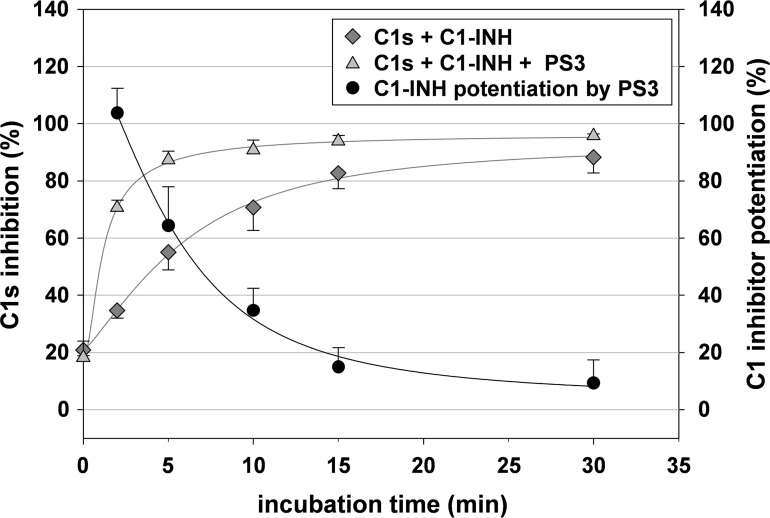
C1s inhibition by C1 inhibitor depends on the incubation time and is accelerated in presence of PS3. C1s was incubated with C1-INH in absence and presence of PS3 (final concentration: 1.25 μg/ml) for 0 to 30 min at 37°C. Then, the residual C1s activity was determined by a chromogenic substrate assay. The C1s inhibition (%) was calculated in relation to the C1s activity in the absence of C1-INH and PS3. The C1-INH potentiation (%) is the increase in C1s inhibition by PS3 in relation to the C1s inhibition by C1-INH alone. Mean ± SD (n ≥ 3 test runs on different days).

To evaluate the velocity of the C1s inhibition by C1-INH and the extent of C1-INH potentiation by SG in dependence on the C1-INH-C1s reaction time, we determined the C1s inhibition after incubation of C1-INH with C1s in absence and presence of PS3 for 0, 2, 5, 10, 15, and 30 min. The inhibition of the amidolytic activity of C1s by C1-INH turned out to be a slow process (Fig 1). After 30 min, it amounted to 88 ± 6% with 50% enzyme inhibition after 4 min. In presence of PS3 (final concentration: 1.25 μg/ml), about 90% C1s inhibition was achieved already after an incubation time of 5 min (Fig 1). Apparently, the reaction between C1-INH and C1s was accelerated in presence of PS3 with 50% enzyme inhibition already after 1 min. However, the calculated C1-INH potentiation related to the C1s inhibition by C1-INH alone decreased with increasing incubation time. After 2 min, PS3 increased the C1s inhibition from 35 ± 3% to 71 ± 3%, corresponding to a potentiation of about 100%, whereas that after 15 min amounted to 15 ± 7%. Although the potentiation after 2 min was even stronger, the following experiments were performed with an incubation time of 5 min for practical reasons. It should be mentioned that the increase of the C1s inhibition from 55 ± 6% to 87 ± 3% by PS3 after 5 min corresponded to a potentiation of 65%, which was nearly the maximal possible one.

### C1s inhibition by C1 inhibitor and its potentiation by sulfated glycans

**Fig 2:**
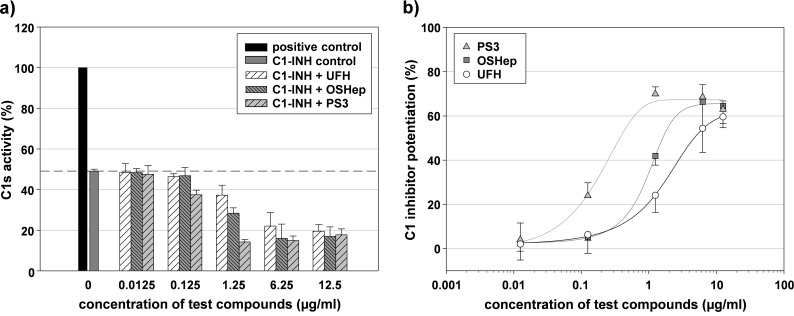
Sulfated glycans potentiate the inhibition of C1s by C1 inhibitor. The C1s activity was measured in the absence and presence of C1-INH and test compounds (final concentration as indicated) by a chromogenic substrate assay. a) Residual C1s activity after incubation with C1-INH in absence and presence of unfractionated heparin (UFH), oversulfated heparin (OSHep) and the β-1,3-glucan sulfate PS3. The grey dashed line marks the C1s activity in presence of C1-INH alone. b) C1-INH potentiation (%) calculated as ratio of the additional C1s inhibition in presence of test compound to the C1s inhibition by C1-INH alone. Mean ± SD (n = 3 test runs on different days).

Before investigating the structure-dependent C1-INH potentiation by SG, we checked whether any of the test compounds has a direct influence on the C1s activity. As none of the tested SG inhibited C1s directly (S1 Fig), the amplified C1s inhibition in the presence of C1-INH was exclusively due to their potentiating effect on C1-INH (Fig 2).

As an example, Fig 2 presents the concentration-dependent reduction of the C1s activity by the three SG UFH, OSHep and PS3 (Fig 2A) and their corresponding C1-INH potentiation (Fig 2B). PS3 was superior to OSHep and UFH; 1.25 μg/ml PS3 led to a C1-INH potentiation of about 65%, which was the highest possible one under the used experimental conditions. To evaluate the C1-INH potentiation by SG in dependence on their structural characteristics, their effects were primarily compared in a final concentration of 6.25 μg/ml.

#### Heparins

**Fig 3:**
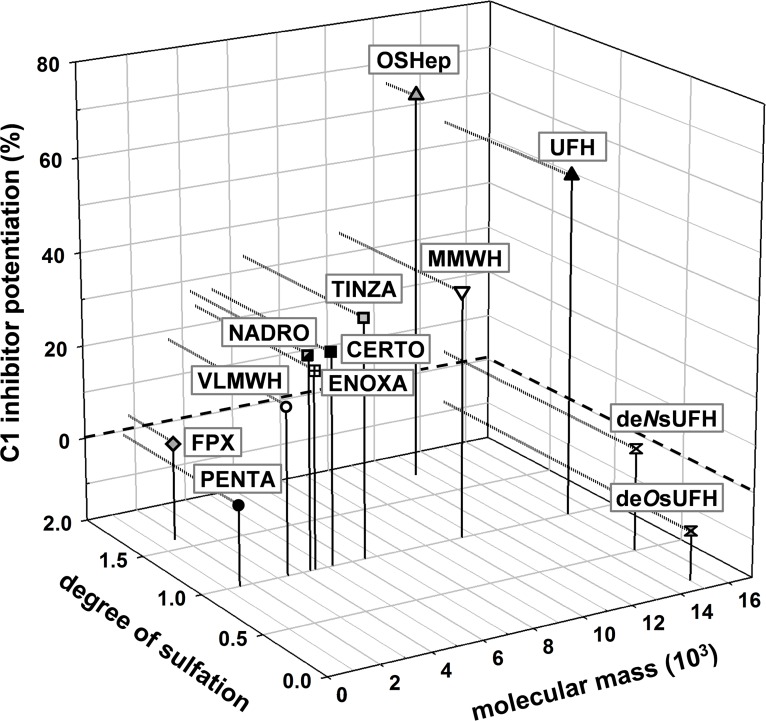
C1 inhibitor potentiation by heparins depends on both their molecular mass and degree of sulfation. The C1s activity was measured in the presence of C1-INH and different heparins (final concentration: 6.25 μg/ml) by a chromogenic substrate assay. The C1-INH potentiation (%) is the increase in C1s inhibition in the presence of test compound in relation to the C1s inhibition by C1-INH alone. Mean of ≥ 3 test runs on different days.

Testing a series of heparins differing in their M_r_ revealed that their C1-INH potentiating effect depends on their M_r_ (S2 Fig). The smallest compound (PENTA, M_r_ 1 500) was completely inactive, whereas UFH was the most active compound among the heparins with similar DS. But the DS showed to play a role as well (Fig 3). Despite their high M_r_, the two desulfated heparins de*N*sUFH and de*O*sUFH were inactive, whereas the highly sulfated pentasaccharide fondaparinux (FPX) exhibited at least a small C1-INH potentiation (3.3 ± 5.3% at a final concentration of 6.25 μg/ml). OSHep led to an even stronger C1-INH potentiation than UFH: about 66% vs. 55% at a final concentration of 6.25 μg/ml (Fig 2, Fig 3).

#### Other glycosaminoglycans

**Fig 4:**
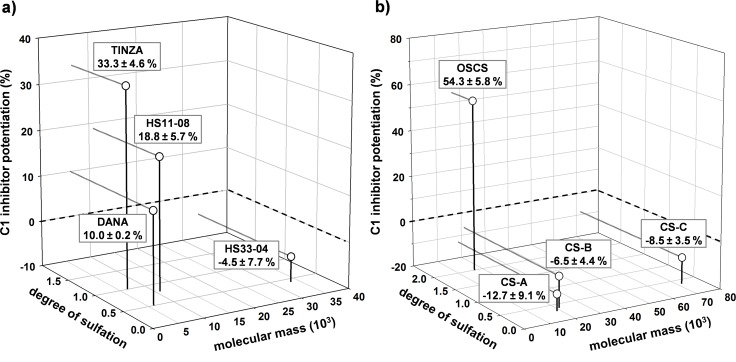
The C1 inhibitor potentiation by other glycosaminoglycans confirms the importance of the degree of sulfation. The C1s activity was measured in the presence of C1-INH and test compounds (final concentration: 6.25 μg/ml) by a chromogenic substrate assay. The C1-INH potentiation (%) is the increase in C1s inhibition in the presence of test compound in relation to the C1s inhibition by C1-INH alone. a) C1-INH potentiation by heparan sulfate species including the LMWH tinzaparin in dependence on their DS and M_r_. b) C1-INH potentiation by chondroitin sulfates in dependence on their DS and M_r_. Mean ± SD (n ≥ 3 test runs on different days).

Among the tested sulfated GAGs, CS-A, CS-B, CS-C and HS33-04, which all have a DS ≤ 0.4, were inactive. In contrast, we found a modest C1-INH potentiating activity for the higher sulfated heparan sulfate HS11-08 (DS 0.8) as well as for danaparoid, which also consists mainly of heparan sulfate. Danaparoid has a mean DS of about 0.5, but additionally contains about 4% high-sulfated antithrombin-binding heparan sulfate [46]. Comparing the three heparan sulfate species (HS33-04, HS11-08 and danaparoid) and heparin (e.g. TINZA), which can be considered as a higher sulfated heparan sulfate, the DS turned out to be the crucial parameter for their C1-INH potentiating activity (Fig 4A). Its important role also becomes obvious with OSCS that was highly active whereas the low-sulfated CS-A, -B and -C rather attenuated the C1s inhibition by C1-INH (Fig 4B). Interestingly, hyaluronic acid (HA, M_r_ 915 000), which is negatively charged due to uronic acids, but not sulfate groups, as well as fragments of HA (oligoHA M_r_ 3 600) slightly potentiated the C1-INH by about 8 ± 2% at a final concentration of 6.25 μg/ml.

#### Dextrans and dextran sulfates

**Fig 5:**
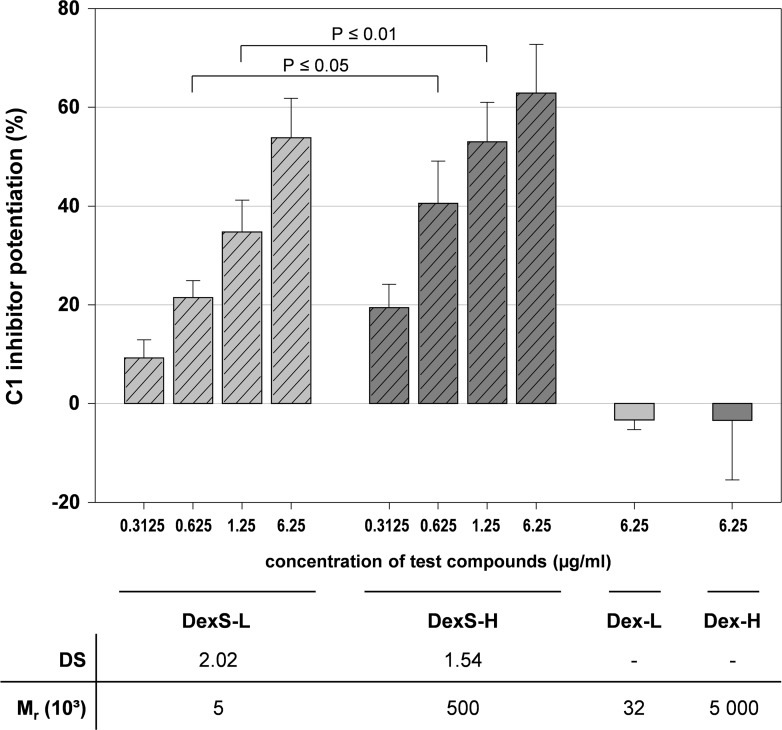
Dextran sulfates enhance the C1s inhibition by C1 inhibitor, whereas the non-sulfated dextrans are inactive. The C1s activity was measured in the presence of C1-INH and dextran sulfates (DexS) or dextrans (Dex) (final concentration as indicated) by a chromogenic substrate assay. The C1-INH potentiation (%) is the increase in C1s inhibition in the presence of test compound in relation to the C1s inhibition by C1-INH alone. Mean ± SD (n ≥ 3 test runs on different days).

To confirm the importance of sulfate groups for the C1-INH potentiation, we tested two dextrans (Dex) and two high-sulfated dextran sulfates (DexS) with different M_r_. Whereas the non-sulfated compounds were inactive, both DexS exhibited a strong C1-INH potentiating effect (Fig 5). DexS-H was more active than DexS-L suggesting that the large difference in their M_r_ exceeds the DS in importance.

#### β-1,3-glucan sulfates

To evaluate the M_r_ and DS dependence of the C1-INH potentiating activity of SG in more detail, we tested two series of semisynthetic β-1,3-glucan sulfates with different DS, namely the low-molecular mass phycarin sulfates (PhyS) and the high-molecular mass curdlan sulfates (CurS).

**Fig 6:**
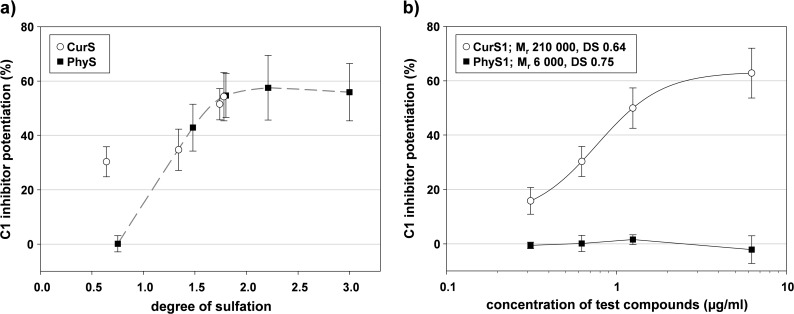
The C1 inhibitor potentiation by β-1,3-glucan sulfates depends on both degree of sulfation and molecular mass. The C1s activity was measured in the presence of C1-INH and test compound (final concentration: a) 0.625 μg/ml b) 0.3125–0.625–1.25–6.25 μg/ml) by a chromogenic substrate assay. The C1-INH potentiation (%) is the increase in C1s inhibition in presence of a test compound in relation to the C1s inhibition by C1-INH alone (CurS = curdlan sulfates; PhyS = phycarin sulfates). Mean ± SD (n = 4 test runs on different days).

These SG potentiated the C1-INH effect against C1s mainly DS-dependently, whereby the effect increased only up to a DS of 1.8 and then reached a kind of plateau (Fig 6A). The influence of the M_r_ was particularly notable in case of SG with relatively low DS: PhyS1 with the lowest DS of 0.75 was inactive whereas the larger CurS1 with an even lower DS of 0.64 exhibited a concentration-dependent C1-INH potentiation (Fig 6B). In contrast to comparable activities at the same gravimetric concentration, testing of approximately similar molar concentrations revealed a considerable activity increase also between the higher sulfated PhyS3 and CurS3a, but not between CurS3a (M_r_ 70 000) and CurS3 (M_r_ 160 000).

#### Algae-derived sulfated polysaccharides and sodium alginate

**Fig 7:**
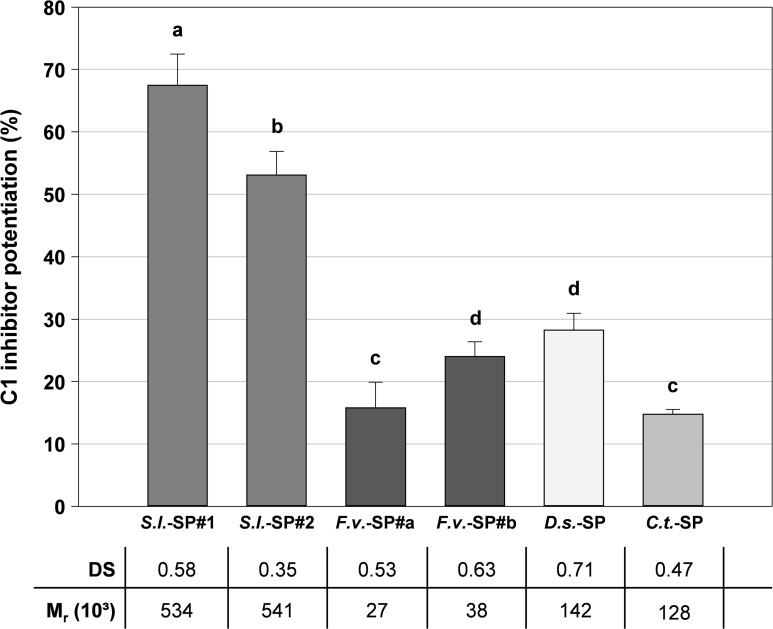
C1 inhibitor potentiation by algae-derived sulfated glycans. The C1s activity was measured in the presence of C1-INH and algae-derived SG from *Saccharina latissima* (*S*.*l*.-SP), *Fucus vesiculosus* (*F*.*v*.-SP), *Delesseria sanguinea* (*D*.*s*.-SP) and *Coccotylus truncatus* (*C*.*t*.-SP) (final concentration: 6.25 μg/ml) by a chromogenic substrate assay. The C1-INH potentiation (%) is the increase in C1s inhibition in the presence of test compound in relation to the C1s inhibition by C1-INH alone. Different letters indicate statistically significant differences (*P* ≤ 0.05). Mean ± SD (n ≥ 3 test runs on different days).

As further examples of SG having glycan structures completely different from GAGs and homoglucans, we examined several sulfated glycans isolated from two red alga and two brown alga species: (1) the sulfated xylogalactan from *Delesseria sanguinea* (*D*.*s*.-SP), (2) the sulfated xylogalactan from *Coccotylus truncatus (C*.*t*.-SP), two batches each of the fucose-containing sulfated polysaccharides from (3) *Saccharina latissima* L. (*S*.*l*.-SP) and (4) *Fucus vesiculosus* L. (*F*.*v*.-SP). The mentioned algae-derived SG are branched heteroglycans with DS of at most 0.71 and M_r_ ranging from 27 000 to 541 000. All these SG enhanced the C1-INH effect against C1s with the high-molecular mass *S*.*l*.*-*SP batches (M_r_ > 500 000) being strongly superior to the other algae-derived SG (Fig 7). The comparison of the *S*.*l*.-SP, *F*.*v*.-SP and xylogalactan batch pairs among themselves again revealed a dependence on the DS. The non-sulfated sodium alginate consisting exclusively of uronic acids was inactive.

Given the observed inactivity of GAGs with DS ≤ 0.42 and the found compensation of a low DS by high M_r_ in the case of CurS, we wanted to check whether the extremely high M_r_ of *S*.*l*.-SP#2 (DS 0.35) is crucial for its pronounced activity. For this, its M_r_ was gradually reduced by hydrothermal depolymerization down to M_r_ 77 000 (Table 2). The C1-INH potentiating effect of these samples in a final concentration of 6.25 μg/ml, however, only decreased at M_r_ ≤ 151 000 and that of the sample with M_r_ 77 000 was reduced by 22% (Table 2) being still higher than that of the sulfated xylogalactans. Analogously, the activity of gradually depolymerized samples of the some higher sulfated *F*.*v*.-SP#b (M_r_ 38 000, DS 0.63) only decreased at M_r_ ≤ 15 900 and was reduced by about 60% at M_r_ 10 300 (Table 2). *C*.*t*.-SP (M_r_ 128 000) and a depolymerized sample (M_r_ 73 000) with an intermediate DS of 0.47 only slightly differed in their C1-INH potentiating activity (Table 2).

**Fig 8:**
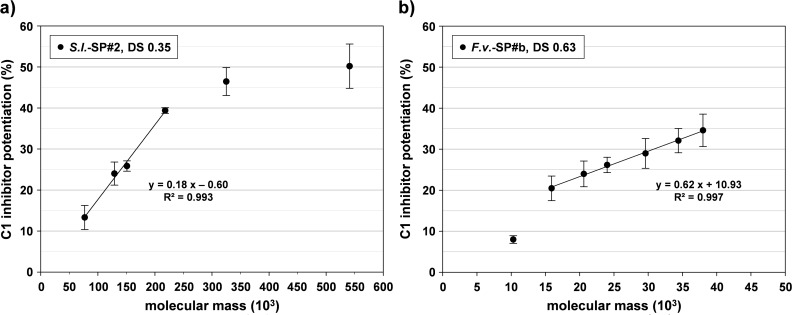
C1 inhibitor potentiation by fucose-containing sulfated glycans from brown algae depends on the molecular mass. The C1s activity was measured in the presence of C1-INH and test compound by a chromogenic substrate assay. The C1-INH potentiation is the increase in C1s inhibition in the presence of test compound in relation to the C1s inhibition by C1-INH alone. a) *S*.*l*.-SP#2 (DS 0.35) in a final concentration of 25 nmol/l and b) *F*.*v*.*-*SP#b (DS 0.63) in a final concentration of 500 nmol/l were tested after depolymerization resulting in M_r_ as indicated on the x-axis. Mean ± SD (n = 4 test runs on different days).

In contrast to the comparison based on gravimetric concentrations, which may underestimate the role of the M_r_, experiments using approximately equal molar concentrations resulted in a stronger M_r_ dependence with biphasic curves for both *S*.*l*.-SP and *F*.*v*.-SP. Whereas the activities of *S*.*l*.-SP samples with M_r_ 541 000 and M_r_ 325 000 did not significantly differ, there was a linear activity decrease at M_r_ ≤ 218 000. The activities of the *F*.*v*.-SP samples linearly decreased in the M_r_ range between 38 000 and 15 900; only the activity decrease between the 15 900- and the 10 300-sample was more pronounced (Fig 8). As confirmed by the direct comparison of the *S*.*l*.-SP and *F*.*v*.-SP samples (100 nmol/l), there was a linear activity decrease in the M_r_ range between 218 000 and 15 900).

Testing both *C*.*t*.-SP samples in the concentration of 50 nmol/l, a C1-INH potentiation by the non-degraded (M_r_ 128 000) and the degraded sample (M_r_ 73 000) amounted to 12.3 ± 2.8% and 9.2 ± 2.0%, respectively. Comparing these activities with those of *S*.*l*.-SP samples with equivalent M_r_ (*S*.*l*.-SP#2_90min_, *S*.*l*.-SP#2_160min_), even 25 nmol/l of the fucose-rich *S*.*l*.-SP were superior.

### FXIIa inhibition by C1 inhibitor and activity modulation by sulfated glycans

C1-INH is not only the major inhibitor of the complement-related C1s, but also neutralizes FXIIa, FXIa, and kallikrein of the contact system. Since FXIIa additionally activates the classical pathway of the complement system by converting C1r to its active form [38], it was of special interest to check, whether sulfated glycans potentiate the FXIIa inhibition by C1-INH, too.

**Fig 9:**
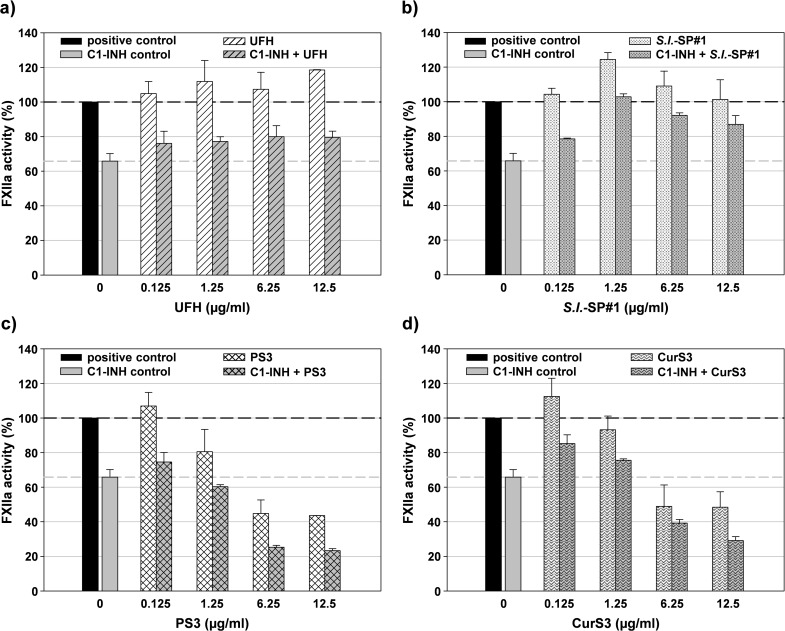
FXIIa inhibition by sulfated glycans in absence and presence of C1 inhibitor. FXIIa was incubated with different sulfated glycans (final concentration as indicated) in absence and presence of C1-INH for 10 min at 37°C. After addition of S-2302 and further incubation for 10 min at 37°C the absorbance at 405 nm was measured. The dark dashed line marks the 100% control in absence of both C1-INH and test compound. The grey dashed line marks the FXIIa activity in presence of C1-INH alone. a) UFH, unfractionated heparin, b) *S*.*l*.-SP#1, sulfated glycan isolated from the brown alga *Saccharina latissima*, c) PS3, low-molecular mass β-1,3-glucan sulfate, d) CurS3, high-molecular mass β-1,3-glucan sulfate. Mean ± SD (n ≥ 2).

To compare the potential activities of SG on the FXIIa inhibition by C1-INH with those on C1s inhibition, an analogous chromogenic assay was used. In line with kinetic data described in literature [28,34], the reaction between FXIIa and C1-INH turned out to be slower than the C1s/C1-INH interaction with 23 min vs. 4 min needed for 50% enzyme inhibition (S3 Fig and Fig 1). Therefore, the incubation time of 5 min in the C1s/C1-INH assay was prolonged to 10 min, which resulted in a FXIIa inhibition of about 30–35% by C1-INH (S3 Fig and Fig 9).

Initial experiments, performed with some SG differing in their glycan structure, namely UFH, *S*.*l*.-SP#1, PS3 and CurS3, revealed divergent effects (Fig 9). The two β-1,3-glucan sulfates PS3 and CurS3 improved the FXIIa inhibition by C1-INH, reaching a C1-INH potentiation of about 124% by 12.5 μg/ml PS3 and about 107% by 12.5 μg/ml CurS3, whereas UFH rather tended to attenuate the FXIIa inhibition by C1-INH. *S*.*l*.-SP led to an even more pronounced attenuation of the FXIIa inhibition, whereby the medium concentration of 1.25 μg/ml completely abolished the C1-INH effect.

Examining the SG in absence of C1-INH resulted in similar direct effects on FXIIa (Fig 9). Whereas UFH only non-significantly improved the FXIIa activity, *S*.*l*.-SP displayed a bell-shaped curve with a significant increase of the FXIIa activity at a concentration of 1.25 μg/ml. In contrast, both β-1,3-glucan sulfates (PS3 and CurS3)–apart from a non-significant activity increase at 0.125 μg/ml–concentration-dependently reduced the FXIIa activity by up to 56% and 52%, respectively (Fig 9C and 9D). To elucidate these divergent findings, we aimed to investigate the structure-dependent effects of SG on the FXIIa activity in more detail.

#### β-1,3-glucan sulfates

**Fig 10:**
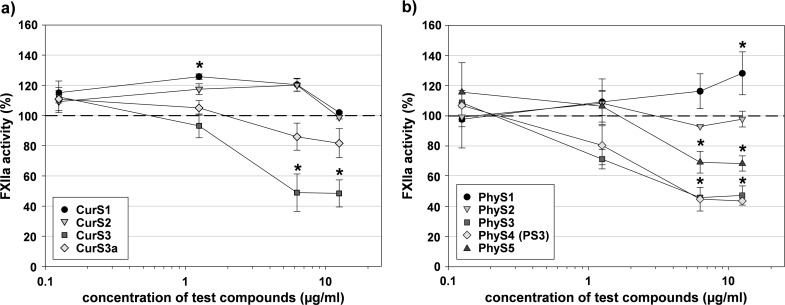
FXIIa activity in presence of β-1,3-glucan sulfates. FXIIa was incubated with a) curdlan sulfates (CurS) or b) phycarin sulfates (PhyS) for 10 min at 37°C. Then, its amidolytic activity was measured using S-2302. The dashed line marks the positive control in absence of test compound. Asterisks indicate statistically significant differences (P≤0.05) compared with the positive control. Mean ± SD (n ≥ 2 test runs on different days).

The high-molecular mass curdlan sulfates displayed opposing effects on the FXIIa activity in dependence on their DS. The lower sulfated compounds CurS1 (DS 0.64) and CurS2 (DS 1.34) improved the FXIIa activity similar to *S*.*l*.-SP, whereby the rather low concentration of 1.25 μg/ml CurS1 caused the strongest increase, i.e. by 25%. As opposed to this, the higher sulfated CurS3 (DS 1.74) and CurS3a (DS 1.78) led to a decrease of FXIIa activity concentration-dependently (Fig 10A).

A similar DS-dependent modulation was observed with the low-molecular mass phycarin sulfates. The PhyS with the lowest DS of 0.75 enhanced the FXIIa activity–in contrast to CurS1 and CurS2 –concentration-dependently, whereas PhyS2 (DS 1.48) had nearly no effect. The three PhyS with DS ≥ 1.80 resulted in a concentration-dependent decrease of FXIIa activity (Fig 10B).

#### Glycosaminoglycans

Like UFH, both the low-molecular weight heparin TINZA and the low-sulfated chondroitin sulfate CS-A (DS 0.30) tended to increase the FXIIa activity, though not significantly. The heparan sulfate HS11-08 (DS 0.80), however, concentration-dependently increased the FXIIa activity up to 141% at 12.5 μg/ml.

**Fig 11:**
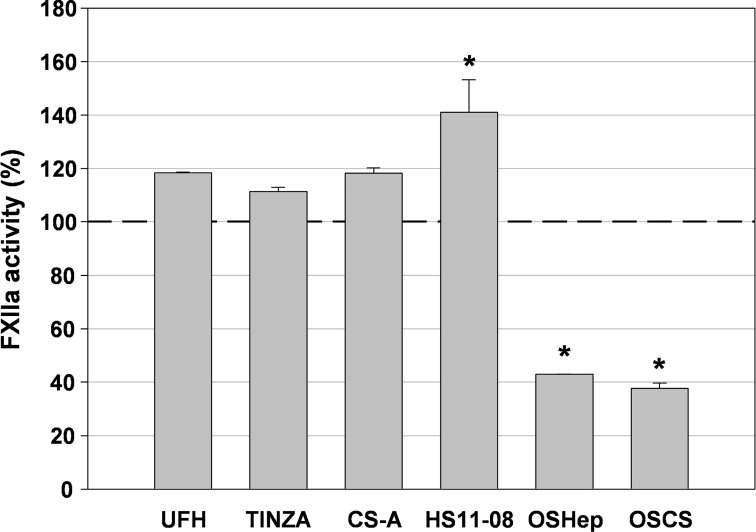
FXIIa activity in presence of glycosaminoglycans. FXIIa was incubated with unfractionated heparin (UFH), tinzaparin (TINZA), chondroitin sulfate A (CS-A), heparan sulfate (HS11-08), oversulfated heparan sulfate (OSHep) and over-sulfated chondroitin sulfate (OSCS) (final concentration: 12.5 μg/ml) for 10 min at 37°C. Then, its amidolytic activity was measured using S-2302. The dashed line marks the positive control in absence of test compound. Asterisks indicate statistically significant differences (P<0.05) calculated with t-test versus positive control. Mean ± SD (n ≥ 2 test runs on different days)

Conversely, the higher sulfated GAGs OSHep (DS 1.75) and OSCS (DS 2.00) concentration-dependently reduced the FXIIa activity by up to about 60% at both 6.25 and 12.5 μg/ml. Fig 11 exemplarily presents the FXIIa activities resulting from incubation with 12.5 μg/ml GAG.

#### Dextran sulfates

**Fig 12:**
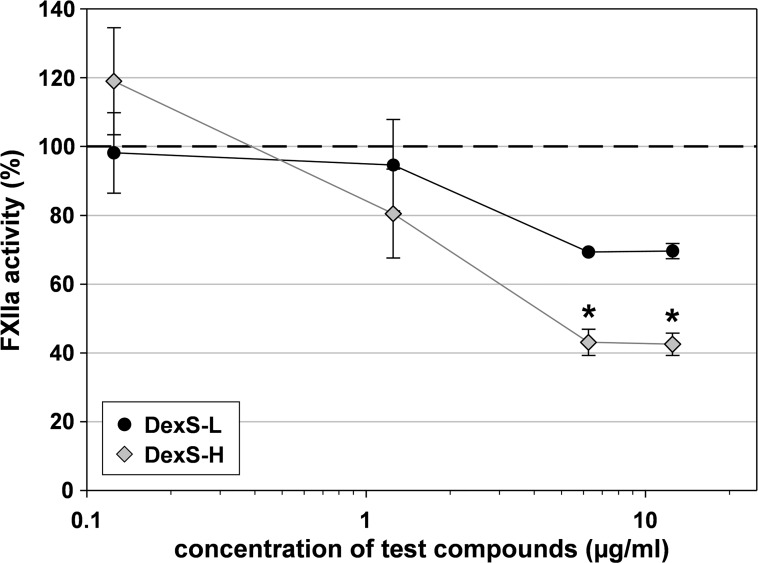
FXIIa activity decreased in presence of dextran sulfate. FXIIa was incubated with two dextran sulfates differing in their molecular mass (DexS-L and DexS-H) for 10 min at 37°C. Then, its amidolytic activity was measured using S-2302. The dashed line marks the positive control in absence of test compound. Asterisks indicate statistically significant differences (P≤0.01) compared with the positive control. Mean ± SD (n ≥ 2 test runs on different days).

Also the two high-sulfated dextran sulfates inhibited the FXIIa activity concentration-dependently, whereby the high-molecular mass DexS-H was superior to the low-molecular mass DexS-L despite its some lower DS (1.54 vs. 2.02) (Fig 12). The maximum inhibition was again reached at 6.25 μg/ml.

## Discussion

The physiological importance of C1-INH, the major regulator of complement and contact system [2], is proven by hereditary and acquired C1-INH-dependent angioedema, which is treated with plasma-derived or recombinant C1-INH [11]. Moreover, C1-INH exhibited therapeutic effects in other diseases and clinical situations [25,24] and may have therapeutic potential in numerous diseases, where the tightly cross-linked complement and contact system are concurrently activated [2]. Due to their C1-INH potentiating effect, sulfated glycans may offer an option to improve either the efficacy of therapeutically applied C1-INH or the activity of endogenous C1-INH. As an elementary step, series of SG were investigated for their C1-INH modulating effect to obtain more information on the structural characteristics of glycans useful for this purpose. Additionally, we examined the effect on a second exemplary serine protease targeted by C1-INH [47,48] to evaluate potential differences concerning the interactions of SG with different enzymes. We have chosen FXIIa, a central enzyme of the contact system, which is additionally involved in complement activation [2].

The following discussion concentrates on a detailed analysis of the found structure-dependent activities to elucidate the respective role of M_r_, DS and glycan structure for the investigated activities of SG.

### C1s, C1 inhibitor and sulfated glycans

#### Kinetics of the reaction between C1s and C1 inhibitor

To focus on the structure-dependent effects of the SG on C1-INH and its target proteases, we used a purified chromogenic substrate assay. In this experimental setting, the C1s inhibition by C1-INH was the stronger the longer the two reactants were incubated together before addition of the C1s substrate (Fig 1). SG such as the β-1,3-glucan sulfate PS3 had no direct inhibitory effect on C1s, but shortened the time for 50% C1s inhibition (1.0 min vs. 4.2 min) with no significant increase in the maximal C1s inhibition. Consequently, C1-INH potentiation by SG actually means an acceleration of this reaction. This mechanism is in accordance with the well-known anticoagulant mode of action of heparins, which catalyze the inhibition of factor Xa and thrombin by antithrombin. However, whereas heparin induces a conformational change of antithrombin (i.e. allostery) and additionally acts as template by binding both antithrombin and thrombin (i.e. bridging), Beinrohr et al. [49] suggested a so-called sandwich-mechanism for the potentiation of C1-INH by polyanions with a heparin binding site in the contact area of the serpin-protease encounter complex. The experiments by Murray-Rust et al. [50] confirmed that polyanion interactions with both C1-INH and C1s play a vital role in the mechanism of accelerating the reaction between C1s and C1-INH and both Rossi et al. [32] and Rajabi et al. [51] proved the binding of heparin to C1-INH as well as C1s by surface plasmon resonance.

#### C1 inhibitor potentiation in dependence on the molecular mass

Heparin was the first compound described to potentiate C1-INH [26,52]. Besides UFH, a number of LMWHs is meanwhile widely used for therapy and prophylaxis of venous thromboembolism. The various LMWHs have different M_r_ profiles [53], but so far it was not known whether this impacts their potentiating effect on the C1-INH-C1s reaction. Testing of four LMWHs and four further heparins representing the M_r_ range of about 1 500 to 35 000 revealed that the C1-INH potentiation by heparins improves with increasing M_r_ and that LMWHs are only half as active as UFH at a rough estimate (S2 Fig). However, based on international units (IU/ml) (LMWHs: ~ 100 IU/mg, UFH: ~ 200 IU/mg), the potentiating activity of LMWHs is similar to that of UFH and at therapeutic plasma concentrations (LMWHs: ~ 1.0 IU/ml; UFH: ~ 0.7 IU/ml), LMWHs may be even superior to UFH. An exception appears to be MMWH (M_r_ 10 500), which showed only a potentiation similar to TINZA (M_r_ 6 500) at equal gravimetric concentrations. This can be explained by their overlapping M_r_ profiles: whereas MMWH contains 30% (m/m) chains with M_r_ < 8 000, the mass percentage (m/m) of TINZA with M_r_ > 8 000 amounts to 22–36%. Moreover, the markedly lower activity of MMWH compared to UFH (M_r_ 15 000) is plausible considering that UFH consists of chains with M_r_ up to 35 000, whereas MMWH contains only 2.5% of chains with M_r_ 12 000-15 000. In contrast to the activity of the heparin oligosaccharides VLMWH (18.2 ± 2.7% C1-INH potentiation at 6.25 μg/ml), which was in line with the results by Rossi et al. [32], the inactivity of the PENTA oligosaccharides (-0.7 ± 2.8% C1-INH potentiation at 6.25 μg/ml) suggests the requirement of a minimum chain length or a minimum number of sulfate groups, respectively. The latter is concluded from the slight C1-INH potentiating effect (3.3 ± 5.3% at 6.25 μg/ml) of fondaparinux (FPX), the synthetic antithrombin-binding pentasaccharide with 8 (DS 1.60) instead of about 5 sulfate groups, which indicates additional relevance of the DS for the C1-INH potentiation. Since pentasaccharides are too short to act as template between serpins and proteases, e.g. antithrombin and thrombin, the slight activity of FPX supports the sandwich theory of Beinrohr et al. [49], but the found M_r_ dependence questions their assumption that the chain length may be of minor importance.

Likewise, already the results of Wuillemin et al. [28] suggest that at least extremely differing M_r_ impact the C1-INH potentiating activity of SG, since they found that high-molecular mass dextran sulfate (M_r_ 500 000, DS 1.9) was significantly more active than a small dextran sulfate (M_r_ 5 000, DS not indicated). This is confirmed by our findings with dextran sulfates: despite its lower DS, DexS-H (M_r_ 500 000, DS 1.54) was still slightly more active than DexS-L (M_r_ 5 000, DS 2.02) (Fig 5). At the level of molar concentrations, DexS-H was even more than two orders of magnitude more active than DexS-L.

The closely linked dependence of the C1-INH potentiating activity of SG on both M_r_ and DS becomes obvious with the two series of β-1,3-glucan sulfates, namely CurS (DS 0.64–1.78) and PhyS (DS 0.75–3.00). The PhyS have a degree of polymerization of about 25 (23–27) and thus a chain length between that of LWMHs and the MMWH, whereas the CurS represent a M_r_ range of 70 000–210 000.

In case of a low DS (< 0.8), a high M_r_ turned out to be crucial for a C1-INH potentiating effect (active CurS1 (DS 0.64, M_r_ 210 000) vs. inactive PhyS1 (DS 0.75, M_r_ 6 000)) (Fig 6B). In contrast, the activity of SG with DS ≥ 1.34, i.e. higher than that of heparins, was independent of the M_r_ (CurS2 vs. PhyS2; CurS3 vs. CurS3a vs. PhyS3) (Fig 6A) or only M_r_-dependent at a molar level, respectively.

In the present study, primarily equal gravimetric concentrations of the SG were compared, since exact molar concentrations cannot be determined due to the inherent polydispersity of glycans. However, this may be associated with an underestimation of the role of the M_r_ as shown by additional experiments using approximately similar molar concentrations. According to these assays, also the activity of SG with DS ≥ 1.34 improved with increasing M_r_ (up to at least M_r_ 70 000). Nevertheless, such a discrepant M_r_-dependence confirms its subordinate role for SG with higher DS. It may partly be due to a changed stoichiometry of the molecular interaction, i.e. one long SG chain may bind to more than one pair of C1-INH/C1s, whereas the binding affinity does not further increase above a certain chain length.

Thus, the C1-INH potentiating activity improves with increasing M_r_ of the SG, whereby its impact becomes crucial, if the DS is below a value in the range of 1.2 as suggested by the data on heparins and β-1,3-glucan sulfates. In this way, a high M_r_ can compensate a low DS. Such a limited importance of the M_r_ is in line with the proposed sandwich mechanism for C1-INH potentiation [49].

The compensatory effect of high M_r_ is confirmed by the algae-derived SG having a DS < 1.0 (Fig 7): The C1-INH potentiation by *S*.*l*.-SP#1 (DS 0.58, M_r_ 534 000) and *S*.*l*.-SP#2 (DS 0.35, M_r_ 541 000) was similar to that of UFH, and that of the other algae-derived SG was in the range of that of the LMWHs. Notably, the high-molecular mass algae-derived SG with DS < 0.5 (*S*.*l*.-SP#2, *C*.*t*.-SP) exhibited activity, whereas the GAGs with DS < 0.5 and M_r_ < 80 000 were inactive (Fig 4).

Moreover, the activity of partially depolymerized fucose-containing sulfated polysaccharides improved with increasing M_r_. But in line with the findings for heparins and β-1,3-glucan sulfates, there is obviously a certain upper limit, which differs depending on both the DS and the basis of comparison (gravimetric vs. molar concentrations) (Fig 8, Table 2). In case of the very low-sulfated *S*.*l*.-SP, a plateau of the activity is only observed at M_r_ > 200 000 (molar comparison).

**Fig 13:**
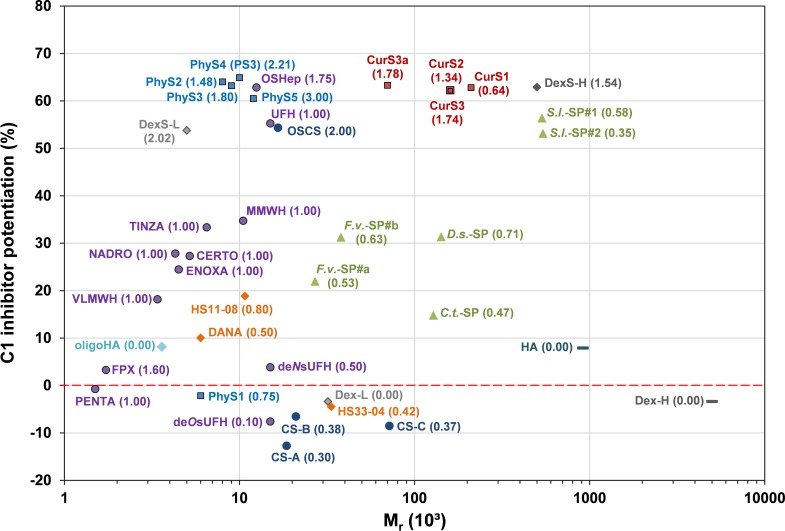
Complete overview of the potentiating activity of the tested sulfated glycans on the C1s inhibition by C1 inhibitor depending on their molecular mass. The C1-INH potentiation (%) by sulfated glycans (final concentration: 6.25 μg/ml) was tested by a chromogenic substrate assay. Numbers in brackets display the compound´s degree of sulfation. The presented data were obtained with the same C1-INH batch, except for *S*.*l*.-SP#2 and *C*.*t*.-SP. Mean of ≥ 3 test runs on different days.

The C1-INH potentiating activity of the tested SG depending on their M_r_ is summarized in Fig 13.

#### C1 inhibitor potentiation in dependence on the degree of sulfation

In line with earlier findings by Wuillemin et al. [28], the inactivity of the two dextrans (Fig 5) showed that the presence of sulfate groups is essential for C1-INH potentiation by glycans. The activities of the compounds within each SG series (heparins, heparan sulfates, chondroitin sulfates, PhyS, *S*.*l*.-SP, *F*.*v*.-SP, sulfated xylogalactans (*D*.*s*.-SP, *C*.*t*.-SP)) revealed a correlation between the DS and the activity (Fig 3, Fig 4, Fig 7). The similar activity of PhyS3 (DS 1.80), PhyS4 (DS 2.21), and PhyS5 (DS 3.00) suggests a certain upper limit for the DS-dependent activity increase, which is however additionally influenced by the M_r_ and possibly by further structural parameters. The minimum DS for any activity turned out to depend on both the M_r_ and the glycan structure and widely varied from 0.34 (*S*.*l*.-SP#2) to 1.6 (FPX) among the tested SG.

Strikingly, some SG with DS < 0.5 (de*O*sUFH, CS-A, CS-B, CS-C, HS33-04) and also PhyS1 (DS 0.75) slightly, but reproducibly attenuated the C1-INH effect against C1s. Already Rent et al. found a reduction of the C1-INH effect by chondroitin sulfate A at concentrations up to 100 μg/ml and only a potentiation at 1000 μg/ml [26]. The more than 10 times higher affinity of heparin to C1-INH (K_D_ 16.7 x 10^−8^ M) compared to C1s (K_D_ 108 x 10^−8^ M) as determined by surface plasmon resonance [32] may provide an explanation for this phenomenon: The charge density of SG with DS < 0.5 may be too low to simultaneously bind to C1-INH and C1s and to form the sandwich complex, but sufficient for the sole binding of the SG to C1-INH resulting in certain impairment of the complex formation between C1-INH and C1s. Dual binding and thus potentiation may only be achieved by either high concentrations or very long SG chains like those of *S*.*l*.-SP.

So far, there is hardly any data whether polyanions other than SG potentiate the C1-INH. Early experiments performed with serum instead of C1-INH revealed a moderate potentiation of C1s inhibition by polyvinyl sulfonate, whereas sodium polyanethol sulfonate (Liquoid^®^, Grobax^®^) neutralized the C1s inhibiting effect of serum [26]. We were able to confirm that the glycan structure is not essential by testing suramin, a symmetric molecule consisting of four benzene units and two naphthalene substituted with six sulfonate groups (M_r_ 1 429). But in light of its moderate activity of about 20% C1-INH potentiation at 6.25 μg/ml (4.37 μmol/l) compared to almost maximum potentiation by only 0.625 μg/ml (0.07 μmol/l) PhyS3, glycan structures may be more suitable to potentiate C1-INH. Polyphosphate, i.e. linear polymers of orthophosphate (NaPO_3_) linked by phosphoanhydride bonds, was reported to bind to C1-INH [54], and just published data demonstrate the C1-INH potentiating activity of polyphosphate consisting of 130 units [55]. Based on the tested gravimetric and molar concentrations its effect was, however, considerably weaker than that of heparin. This is in line with our preliminary data on curdlan phosphate (-2.4 ± 1.8% C1-INH potentiation at a final concentration of 6.25 μg/ml) indicating that phosphate groups might be less prone than sulfate groups to build the sandwich complex for potentiation. Similarly, uronic acids, which constitute 50% of the monosaccharides of the GAGs, seem to make only a small contribution to the C1-INH potentiation as concluded from the slight potentiating activity by both HA and oligoHA. The apparently contradictory effect of sodium alginate (-5.1 ± 1.1% C1-INH potentiation at a final concentration of 6.25 μg/ml) could be explained by its ordered conformation due to complex formation (egg-box model) in the presence of Ca^2+^ and Mg^2+^ [56], which may prevent sandwich complex formation.

**Fig 14:**
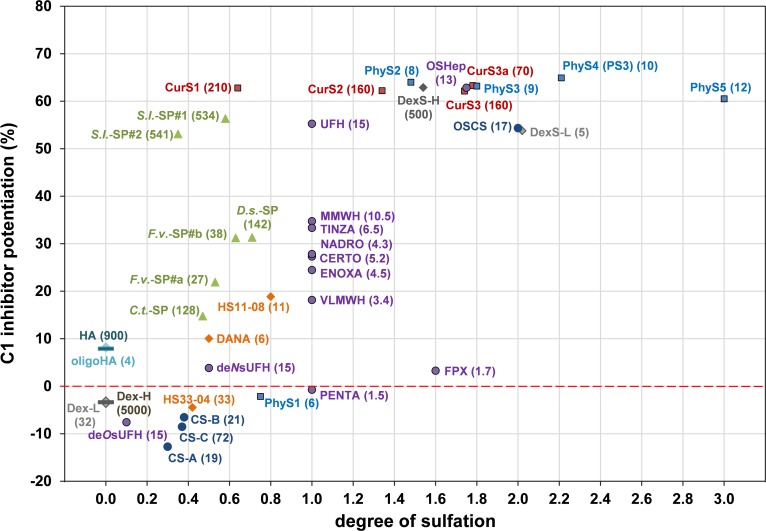
Complete overview of the potentiating activity of the tested sulfated glycans on the C1s inhibition by C1 inhibitor depending on their degree of sulfation. The C1-INH potentiation (%) by sulfated glycans (final concentration: 6.25 μg/ml) was tested by a chromogenic substrate assay. Numbers in brackets display the compound´s molecular mass, M_r_ (10^3^). The presented data were obtained with the same C1-INH batch, except for *S*.*l*.-SP#2 and *C*.*t*.-SP. Mean of ≥ 3 test runs on different days.

The C1-INH potentiating activity of the tested SG depending on their DS is summarized in Fig 14.

#### C1 inhibitor potentiation in dependence on the glycan structure

The complexity of Fig 13 and Fig 14 illustrates that the C1-INH potentiating activity of the tested SG does not only depend on their DS and M_r_, but also on their glycan structure. The comparison of SG with different structures, but either similar activity or appropriate DS and M_r_ (Table 3) suggests the following effects of the glycan structure on the C1-INH potentiating activity: (1) OSHep was more active than OSCS despite its some lower DS and M_r_. This may be due to the high iduronic acid content of heparins, whereas chondroitin sulfates exclusively contain glucuronic acid. A unique feature of iduronic acid is its conformational flexibility, which is considered to be advantageous for interactions with biomolecules [57]. (2) CurS1 was as active as DexS-H having a 2.4 timer higher DS and PhyS2 (DS 1.48) was more active than DexS-L (DS 2.02) so that a linear β-1,3-glucan structure seems to be more suitable to form sandwich complexes than the highly branched α-1,6-glucan structure of dextrans. (3) As indicated by the superiority of PhyS2 and PhyS3 over OSHep, the β-1,3-glucan structure is supposed to be also more favourable than that of heparins, whereby this could also be due to their homogenous composition, whereas heparins consist of a complex mixture of heterogeneous molecules [57]. (4) Among the algae-derived SG, the fucose-containing sulfated polysaccharides (syn. fucoidans) from brown algae turned out to represent better C1-INH modulators than xylogalactans from red algae. This was shown by the higher activity of the low-sulfated, depolymerized *S*.*l*.-SP#2 samples compared to *C*.*t*.*-*SP and *D*.*s*.-SP as well as by *F*.*v*.-SP#b, which was as active as *D*.*s*.-SP despite its lower M_r_ and DS (Table 3)_._

**Table 3:** Direct comparison of the C1 inhibitor potentiation by test compounds with differing glycan structure.

Test compound	M_r_^1^	DS^2^	C1 inhibitor potentiation (%)^3^
OSHep	12 500	1.75	62.8 ± 10.2
OSCS	16 600	2.00	54.3 ± 8.5
CurS1	210 000	0.64	62.8 ± 9.1
DexS-H	500 000	1.54	62.9 ± 9.8
PhyS2	8 000	1.48	64.0 ± 13.2
DexS-L	5 000	2.02	53.8 ± 8.0
PhyS2	8 000	1.48	65.1 ± 1.5 ^a^
PhyS3	9 000	1.80	71.6 ± 2.0 ^a^
OSHep	12 500	1.75	45.6 ± 8.6 ^a^
*S*.*l*.-SP#2_90 min_	129 000	0.35	45.7 ± 2.1
*C*.*t*.-SP	128 000	0.47	14.8 ± 0.7
*D*.*s*.-SP	142 000	0.71	28.3 ± 2.7
*F*.*v*.-SP#b	38 000	0.63	24.0 ± 2.4
*D*.*s*.-SP	142 000	0.71	28.3 ± 2.7

^1^ relative molecular mass; determined by size exclusion chromatography with a MALLS detector; the indicated molecular mass is the weight averaged M_r_ (M_w_)

^2^ degree of sulfation (sulfate groups per monosaccharide in relation to the absolute glycan content) calculated from sulfur content (elemental analysis)

^3^ test compounds in a final concentration of 6.25 μg/ml unless stated otherwise; mean ± SD, n = 4 test runs on different days

^a^ test compounds in a final concentration of 1.25 μg/ml due to not-significant differences at 6.25 μg/ml; mean ± SD of one duplicate measurement on a day with direct comparison of these compounds

### FXIIa, C1 inhibitor and sulfated glycans

#### State of research

The tested SG displayed highly divergent effects on the C1-INH-C1s interaction ranging from certain C1-INH neutralization up to potentiation stronger than that by dextran sulfates, which have so far been described as the most potent compounds [28]. Therefore, the question arose how these SG modulate the interactions of C1-INH with other serine proteases.

Of these, FXIIa, appeared to be the most interesting one, because it initiates not only contact activation, but also triggers the C1-mediated complement activation [2,58,38] and is predominantly inhibited by C1-INH like C1s [47,48]. Moreover, the influence of GAGs on the C1-INH-FXIIa reaction [36,34] was found to differ from that on C1-INH-C1s [28], C1-INH-FXIa [34,35] and C1-INH-kallikrein [37,34]. GAGs and dextran sulfates (in the order dextran sulfate > heparin > heparin sulfate; low-molecular mass dextran sulfate > LMWHs > dermatan sulfate) strongly accelerated the inhibition of FXIa similar to that of C1s [34,35] and dextran sulfate increased the FXIa-inhibition by C1-INH in plasma from 44% to 99%, whereas all these SG did not significantly modify the kallikrein activity [34,37]. The inactivation of FXIIa by C1-INH was also not influenced by heparins, heparan sulfate and dermatan sulfate, but, on the contrary, some polyanions (high- and low-molecular mass dextran sulfate, an extremely high heparin concentration (400 μg/ml), kaolin and sulfatides) were reported to protect FXIIa from inhibition by C1-INH [36,34,35].

#### Direct FXIIa activity modulation independent of C1 inhibitor

In part, our initial results seemed to confirm the distinct effects of SG on the FXIIa inhibition by C1-INH, namely no significant change by UFH and attenuation of the C1-INH activity by the very-high-molecular mass *S*.*l*.-SP up to complete recovery of FXIIa activity. Strikingly, however, the semi-synthetic β-1,3-glucan sulfates PS3 and CurS3 apparently potentiated C1-INH. Ultimately, this unique activity was due to direct inhibition of the amidolytic activity of FXIIa so that the improved FXIIa inhibition by C1-INH resulted from an additive effect. To our knowledge such a direct influence on FXIIa was not observed before. So far, dextran sulfate and GAGs have only been described to directly affect the amidolytic activity of both kallikrein and FXIa [34,35,59] and to reduce also the catalytic activity of kallikrein on the hydrolysis of plasminogen [37].

Moreover, the reduction of the FXIIa inhibition by C1-INH in the presence of *S*.*l*.-SP and other SG was shown to be not due to protection of FXIIa from inactivation as previously suggested [36,34,28] but due to direct stimulation of its amidolytic activity.

Beinrohr et al. [49] proposed that the C1-INH potentiating activity of polyanions correlates with the positive charges on the contact site of the serine proteases. Accordingly, the most significant effect is therefore observed with FXIa carrying the most positive charges, whereas the strikingly acidic FXIIa prevents the formation of the sandwich complex and even impairs the inactivation by C1-INH. However, this hypothesis conflicts not only with our observations of direct effects of SG on the FXIIa activity, but also with the identified binding site of FXII for negatively charged surfaces on the fibronectin type II domain of FXII, in particular residues 39 to 47 [60], and the found binding of OSCS on both FXII and FXIIa [61].

#### Structure-dependent FXIIa activity modulation by sulfated glycans

Although the mode of action of SG on FXIIa needs further investigation, e.g. studies in a more physiological system, testing of a considerable number of SG provided the following information about structure-activity relationships.

The type of modulation was decisively determined by the DS: (1) All the tested SG with DS ≥ 1.35 inhibited FXIIa with an optimum at DS 1.8–2.2, whereby maximum inhibition was observed at 6.12 μg/ml and did not exceed about 60%. (2) Heparins, i.e. SG with a DS of about 1.0, did not significantly modulate the FXIIa activity. (3) All the tested SG with DS ≤ 0.80 stimulated the FXIIa activity by up to about 40% reached by HS11-08 (DS 0.80, M_r_ 10 800) and *S*.*l*.-SP#1 (DS 0.58, M_r_ 534 000).

Besides the DS, the M_r_ of the SG showed to play a role. The FXIIa stimulating SG with Mr ≤ 150 000, like HS11-08, exhibited a concentration-dependent activity increase (highest concentration 12.5 μg/ml), whereas larger ones, e.g. *S*.*l*.-SP#1, displayed a bell-shaped curve with maximum stimulation at 1.25 μg/ml. In the case of the FXIIa inhibiting high-sulfated compounds, those with high M_r_ (DexS-H, CurS) showed a stimulating effect at the lowest concentration (0.125 μg/ml).

Overall, only high-sulfated SG with a limited M_r_ like PhyS clearly inhibited FXIIa up to a certain extent, whereas both low-sulfated SG and large SG seem to be able to exhibit FXIIa stimulating effects in dependence on their concentration.

Negatively charged surfaces are well-known to induce auto-activation of FXII, but a stimulating effect on the FXIIa activity has not been reported so far. Notably, a recent study demonstrated that FXII, in the presence of polyphosphates (polyP) (scFXII-polyP70), develops the ability to hydrolyze a chromogenic substrate as well as its natural substrates FXI and prekallikrein without becoming a two-chain enzyme and thus FXIIa [62]. Binding of polyP showed to be essential for this activity and a bell-shaped concentration response to polyP was evident, as is typical of surface-mediated reactions. Accordingly, it may be conceivable that also the binding of SG to FXIIa may induce or stabilize the conformation of FXIIa optimal for its enzymatic activity.

What impact the observed direct effects of SG on the activity of FXIIa have in more physiological systems, still needs to be investigated. This is difficult to estimate due to the (patho-)physiological complex role of FXII/FXIIa (coagulation, prekallikrein (bradykinin generation) and complement activation), its regulation by several endogenous inhibitors [47] and the multivalent interactions of SG with involved enzymes and inhibitors. Moreover, SG may induce autoactivation of FXII [63] (e.g. PhyS ≤ heparins < OSCS < DexS (unpublished data)). Consequently, it is very challenging to assess the overall effect of SG by any *in vitro* assays. This is illustrated by the OSCS, misused to falsify heparin, which exhibits both anti- and procoagulant and both complement activating and inhibiting effects *in vitro* [61,64,30]. The adverse clinical events observed with OSCS-contaminated heparin have been linked with the activation of kinin-kallikrein and complement pathways [64]. However, the majority of patients who received contaminated heparin did not experience an adverse event. To clarify this fact, a study was performed including the analysis of patient data and plasma samples [65]. It revealed that low C1-INH levels turned out as a risk factor for adverse events, whereas high levels were suggested to be protective, which once again emphasizes the importance of an appropriate C1-INH function.

## Conclusion

In summary, the evaluated structure-activity relationships indicate that the potentiating effect of SG on the C1s inhibition by C1-INH correlates with their DS and M_r_, but is additionally influenced by their glycan structure. The dependence on DS and M_r_ are closely linked, which means that the M_r_ plays especially an important role, if the DS is below ~ 1.2. Both the minimum M_r_ needed for any potentiation by low-sulfated SG and the upper M_r_ limit still having an impact, increases with decreasing DS of the SG. Accordingly, SG with a very low DS require a high M_r_ to exhibit any C1-INH potentiation, whereas for SG with a very high DS a chain length of only 5 monosaccharides (FPX) turned out to be sufficient for a slight effect. These observations are consistent with the sandwich mechanism, whereby the polyanion interposes as a negatively charged bridge between positively charged surfaces of C1-INH and C1s [50,49]. Among the glycan structures examined in this *in vitro* study, both β-1,3-glucans as well as fucose-rich glycans turned out to be favourable structures for the C1-INH potentiation.

In contrast to the C1-INH-C1s interaction, SG do not influence the C1-INH-FXIIa interaction, but SG with DS and M_r_ considerably differing from that of heparins can directly modulate the FXIIa activity. Whereas SG with DS < 1.0 turned out to stimulate the FXIIa activity in a bell-shaped manner, SG with DS > 1.0 concentration-dependently inhibited the FXIIa activity up to a certain extent. However, especially in the case of FXIIa with its complex functions and regulation, SG may additionally modulate other enzymes and inhibitors involved in this network. Further investigations are, therefore, needed, whereby the heparin contaminant OSCS demonstrated that the finally resulting *in vivo* effect is hardly conceivable from *in vitro* studies.

As usual in drug development, an essential precondition for the use of a SG as C1-INH amplifier is its safety. In this regard, a corresponding SG should neither stimulate FXIIa nor trigger the FXII auto-activation as it is known from polyanions with high M_r_ [63]. Since SG usually act as multivalent biomodulators [53] and can exhibit additional activities in dependence on their structural parameters, the overall pharmacological profile has to be considered. Generally, SG appropriate for use as C1-INH amplifiers should be less anticoagulant than heparins. Additional anti-inflammatory activities are assumed to be beneficial. Given the reduced adverse effects (e.g. heparin-induced thrombocytopenia) [66] and improved pharmacokinetics of LMWHs compared to UFH [53], a rather low M_r_ is supposed to be advantageous for *in vivo* applicable SG.

Taking into account these aspects as well as the results of the present study, PhyS, i.e. low-molecular mass β-1,3-glucan sulfates, may represent promising candidates for further investigations. Compared to heparins and other GAGs, PhyS have the advantage that they are chemically defined, semi-synthetic glycans of non-animal origin. Though PS3 proved to have pronounced *in vivo* anti-inflammatory effects [42,67], a PhyS with a lower DS may be more appropriate for use as C1-INH amplifier. Further, depolymerized, sulfated fucan-rich glycans seem to be worth to be evaluated as lead structures in more detail.

In conclusion, the results of this study represent basic pharmacological information, which may be useful for the initial steps towards developing C1-INH potentiating compounds for improving the current C1-INH replacement therapy or enhancing the potency of endogenous C1-INH in diseases and clinical situations characterized by inappropriate activation of complement and contact system.

## Supporting Information

S1 Fig Sulfated glycans do not inhibit C1s esterase directly. The activity of C1s in the absence (= 100% activity) and presence of test compounds (final concentration: 6.25 μg/ml) was measured by a chromogenic substrate assay. Mean ± SD (n ≥ 3 test runs on different days).
(TIF)

S2 Fig C1 inhibitor potentiation by heparins with similar degree of sulfation depends on their molecular mass. The C1s activity was measured in the presence of C1-INH and different heparins (final concentration: 6.25 μg/ml) by a chromogenic substrate assay. The C1-INH potentiation (%) is the increase in C1s inhibition in the presence of test compound in relation to the C1s inhibition by C1-INH. Mean ± SD (n ≥ 4 test runs on different days).
(TIF)

S3 Fig FXIIa inhibition by C1 inhibitor in dependence on the incubation time. FXIIa was incubated with C1-INH for 0 to 60 min at 37°C and its amidolytic activity measured using S-2302. The FXIIa inhibition (%) was calculated in relation to the FXIIa activity in the absence of C1-INH alone. Mean ± SD (n ≥ 3 test runs on different days).
(TIF)
