# Supplementary figures and images for: Regulation of Complement and Contact System Activation via C1 Inhibitor Potentiation and Factor XIIa Activity Modulation by Sulfated Glycans – Structure-Activity Relationships
Source: PLoS One. 2016 Oct 26;11(10):e0165493. doi: 10.1371/journal.pone.0165493 (PMC5082678; doi:10.1371/journal.pone.0165493)

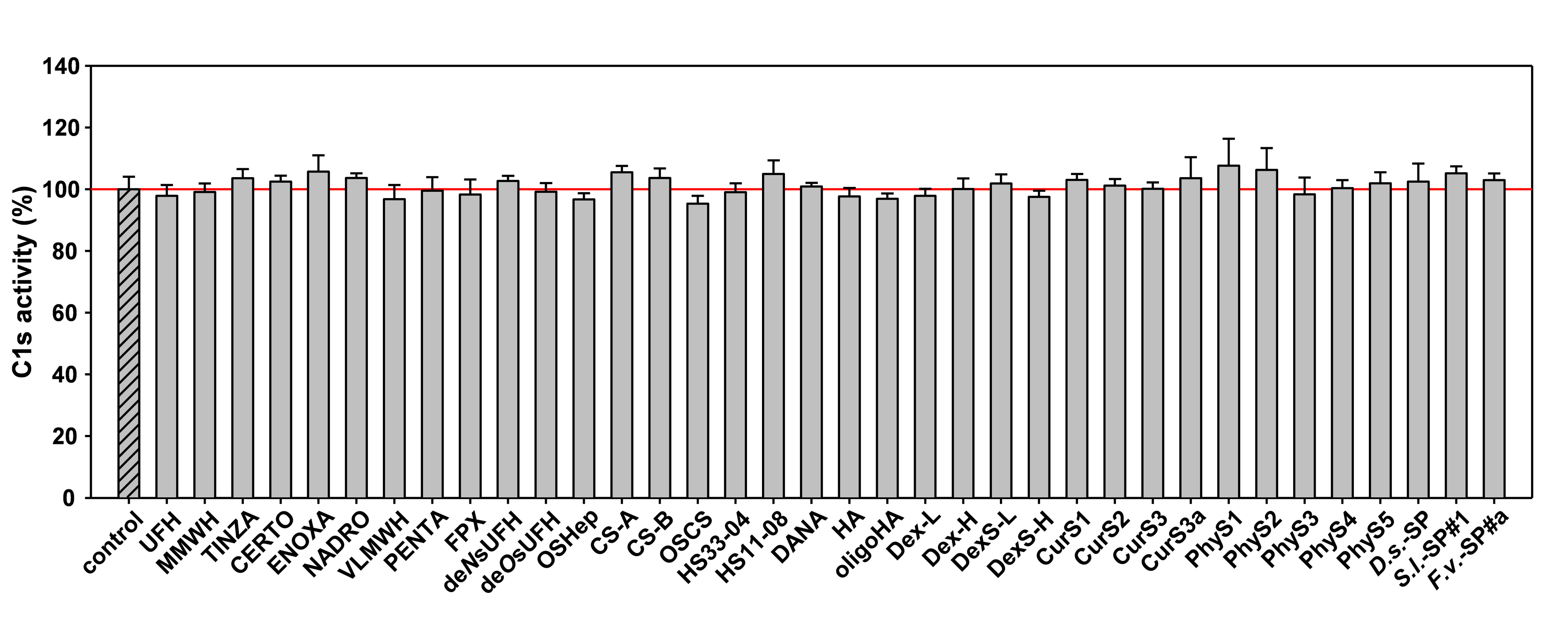

Supplement: S1 Fig — The activity of C1s in the absence (= 100% activity) and presence of test compounds (final concentration: 6.25 μg/ml) was measured by a chromogenic substrate assay. Mean ± SD (n ≥ 3 test runs on different days). (TIF) [file pone.0165493.s001.tif]

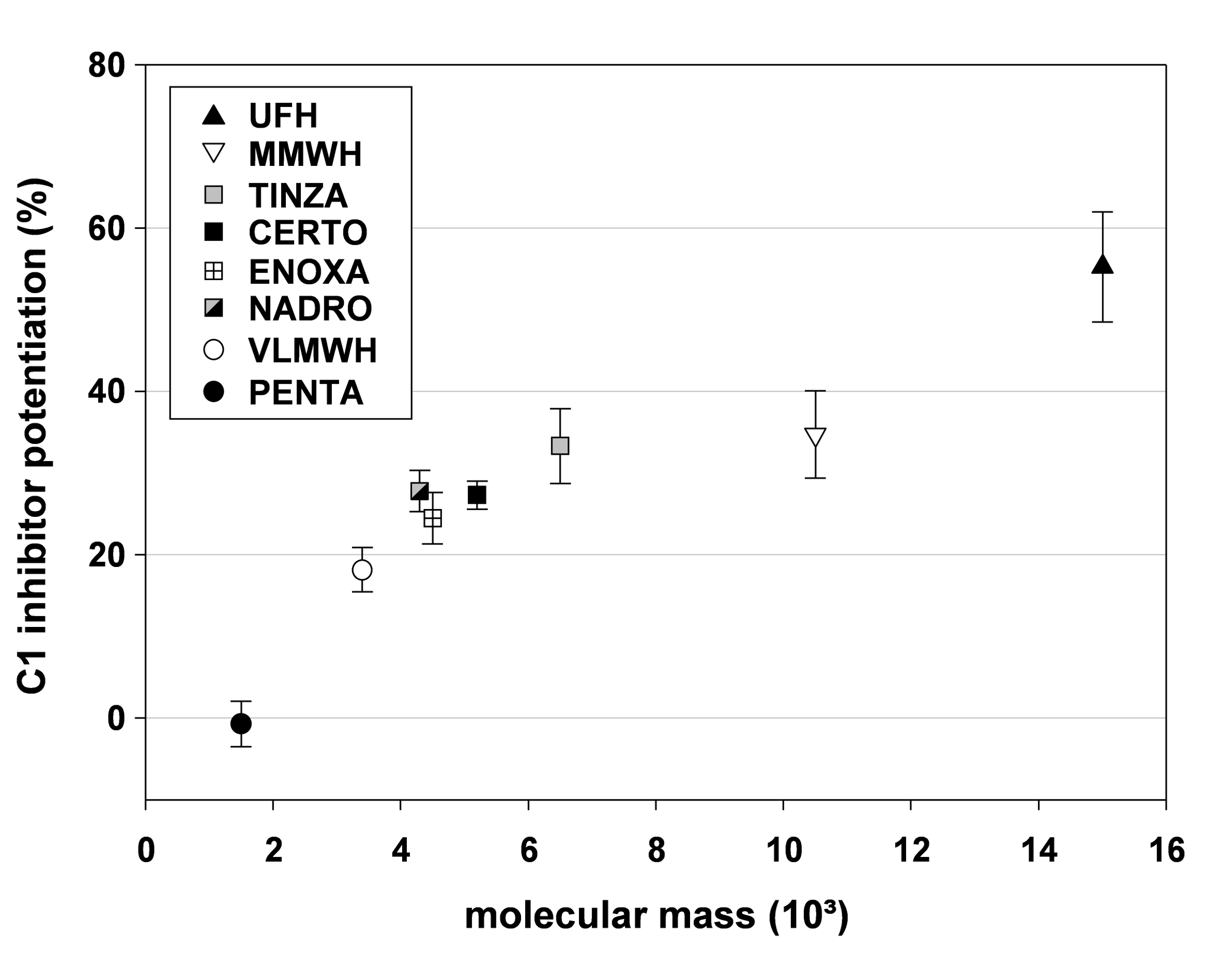

Supplement: S2 Fig — The C1s activity was measured in the presence of C1-INH and different heparins (final concentration: 6.25 μg/ml) by a chromogenic substrate assay. The C1-INH potentiation (%) is the increase in C1s inhibition in the presence of test compound in relation to the C1s inhibition by C1-INH. Mean ± SD (n ≥ 4 test runs on different days). (TIF) [file pone.0165493.s002.tif]

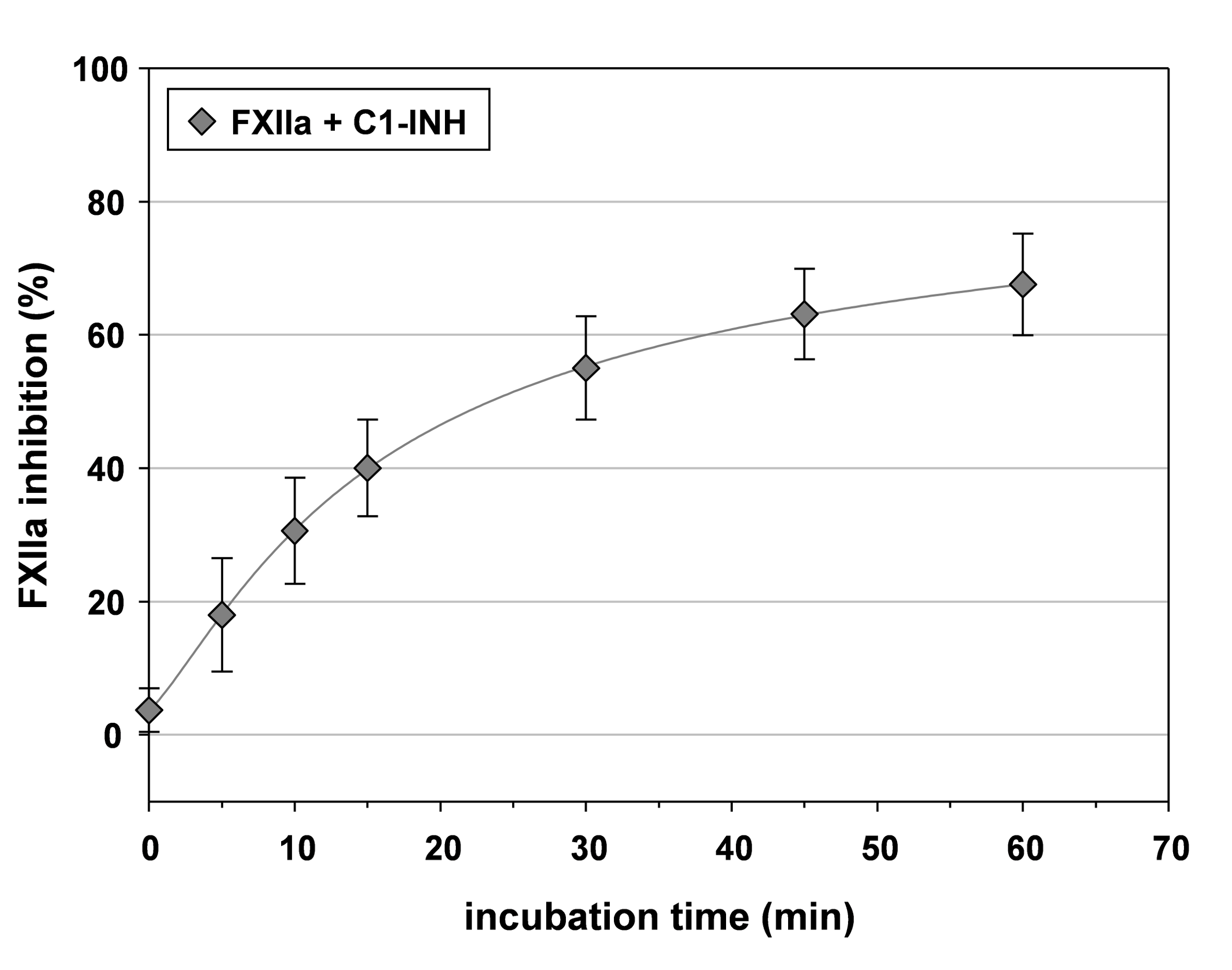

Supplement: S3 Fig — FXIIa was incubated with C1-INH for 0 to 60 min at 37°C and its amidolytic activity measured using S-2302. The FXIIa inhibition (%) was calculated in relation to the FXIIa activity in the absence of C1-INH alone. Mean ± SD (n ≥ 3 test runs on different days). (TIF) [file pone.0165493.s003.tif]
